# Achieving efficient violet-blue electroluminescence with CIE_*y*_ <0.06 and EQE >6% from naphthyl-linked phenanthroimidazole–carbazole hybrid fluorophores†
†Electronic supplementary information (ESI) available. See DOI: 10.1039/c6sc05619a


**DOI:** 10.1039/c6sc05619a

**Published:** 2017-02-20

**Authors:** Wen-Cheng Chen, Yi Yuan, Shao-Fei Ni, Qing-Xiao Tong, Fu-Lung Wong, Chun-Sing Lee

**Affiliations:** a Center of Super-Diamond and Advanced Films (COSDAF) , Department of Chemistry , City University of Hong Kong , Hong Kong SAR , PR China . Email: apcslee@cityu.edu.hk; b Department of Chemistry and Key Laboratory for Preparation and Application of Ordered Structural Materials of Guangdong Province , Shantou University , 243 University Road , Shantou , Guangdong 515063 , PR China . Email: qxtong@stu.edu.cn; c Department of Chemistry , Southern University of Science and Technology , Shenzhen , 518055 , PR China

## Abstract

In this work, we revealed a new approach for the development of efficient violet-blue emitting materials featuring a hybrid local and charge transfer (HLCT) excited state through the incorporation of naphthyl group(s) as a weak n-type π spacer in a donor–π–acceptor (D–π–A) system. The resulting materials (TPINCz and TPIBNCz) show improved intramolecular charge transfer properties and highly efficient violet-blue fluorescence. It is demonstrated that the pattern of the π spacers has significant influence on the photophysical properties. The incorporation of a naphthyl/binaphthyl spacer between the donor and acceptor moieties can alleviate the common dilemma that enhancing device performance by increasing the charge transfer excited properties often leads to red-shifted emissions. A device using TPINCz as an emissive dopant shows a violet-blue emission with CIE coordinates of (0.153, 0.059) and a record high EQE of 6.56 ± 0.11% at a brightness of 1000 cd m^–2^. To the best of our knowledge, this performance is the highest among the reported devices with CIE_*y*_ ≤0.08. Our study provides a new pathway for the design of high-performance violet-blue emitters with a D–π–A architecture in organic electroluminescence applications.

## Abstract 2

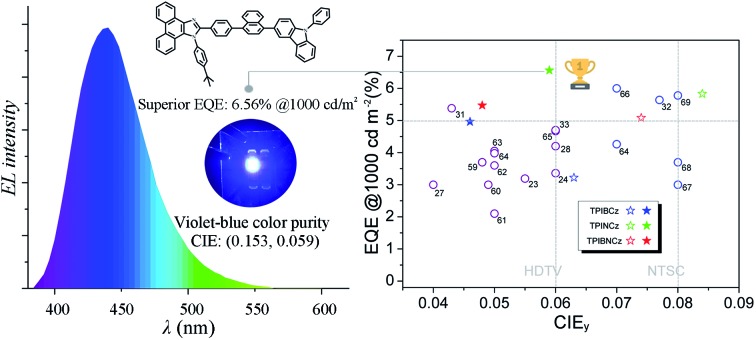
Naphthyl-linked donor–π–acceptor fluorophores were utilized to achieve high performance and good color purity violet-blue emission in organic light-emitting devices (OLEDs).

## Introduction

The dramatic progress in the area of organic optoelectronics, observed over the last couple of decades, has been largely realized by the successful advance in numerous conjugated organic materials, showing a wide range of tunable electrical/photo-physical characteristics.^1^–^7^ Understanding the structure–property relationship and mastering the ways to manipulate these unique properties significantly lead to a rapid development in organic semiconductor based applications, such as organic field-effect transistors, photovoltaic and electroluminescence (EL) devices *etc.* In the area of light generation, considerable interest in organic light-emitting devices (OLEDs) is derived from their attractive prospects as a new generation of full-color flat panel display and solid-state lighting technologies.^8^–^10^ Efficient violet-blue EL is very important in OLEDs since violet-blue fluorophores can be employed as energy donors to generate light covering the entire visible region and white light emission.^11^–^13^ Additionally, efficient short-wavelength EL plays a key role in reducing the power consumption and expanding the color gamut of full-color displays.^14^,^15^ OLEDs with short-wavelength emission also have promising applications in other fields, such as chemical and biological sensing,^16^ phototherapy,^17^ photocopying,^18^ high-density information storage,^19^ and sterilization^20^*etc.* Therefore, the development of high-performance violet-blue light-emitting materials is crucial for the burgeoning OLED technology.

However, unlike their red, green and sky-blue counterparts with desirable performances, to date, reports on efficient organic fluorophores with deep-blue or violet-blue emission in the Commission Internationale de L’Eclairage (CIE) map that match or surpass the high-definition television (HDTV) standard blue index (CIE coordinates of (0.15, 0.06)) are rare.^21^,^22^ In principle, violet-blue emission requires materials with a wide optical gap, which implies a large HOMO (highest occupied molecular orbital)–LUMO (lowest unoccupied molecular orbital) offset, leading to inferior carrier injections. Moreover, the corresponding molecular conjugation must be strictly controlled. This often in turn results in a drop in the photoluminescence quantum yield (*Φ*_f_) and poor electrical properties. These also cause difficulty in the molecular design and synthesis for better thermal and morphological stability.^23^,^24^

In the past decade, much effort has been devoted to the exploitation of a wide range of blue fluorescent dyes. For instance, Gao *et al.* developed a phenanthro[9,10-*d*]imidazole (PI) derivative and applied it in a violet-blue OLED exhibiting CIE coordinates of (0.166, 0.056), but the maximum external quantum efficiency (EQE) was only 3.02%.^25^ Zhang *et al.* reported a blue emitter TBPMCN that can utilize ∼100% excitons in a non-doped device.^26^ This OLED showed high performance with an EQE_max_ of 7.8% and a sky-blue EL emission (CIE coordinates of (0.16, 0.16)). An effective violet-blue OLED based on a silicon containing compound SiPIM was reported with an EQE of 6.29% and a superior color index of (0.163, 0.040).^27^ Unfortunately, the non-conducting silicon molecular framework of SiPIM leads to a high turn-on voltage (*V*_on_, 4.2 V) and low power efficiency (PE). So far, the most effective violet-blue OLED was from a donor–acceptor (D–A) molecule with bisanthracene as the emitting core, showing an EQE up to 12% and a color purity of (0.15, 0.06).^28^ It is suggested that triplet fusion (TF) is responsible for such a high EL performance. Another efficient deep-blue OLED reported by Adachi’s group employed a sulfone/carbazole hybrid emitter that can emit thermally activated delayed fluorescence (TADF) with a maximum EQE of 9.9% and CIE coordinates of (0.15, 0.07).^29^ Very recently, Hatakeyama and co-workers designed an ultrapure blue TADF-based emitter, DABNA-1; its HOMO–LUMO can be separated inter-atomically, which endows the corresponding device with a high EQE of 13.5% and narrow blue EL spectra with CIE coordinates of (0.13, 0.09).^30^ Nevertheless, the high efficiencies from the above-mentioned devices suffered from serious efficiency roll-off at high luminances (*e.g.* 1000 cd m^–2^), resulting in compromised performance at applicable brightness. To date, there are few available reports for highly efficient deep-blue OLEDs matching or approaching the HDTV standard with slow efficiency roll-off, except for a handful of recent studies in the literature.^31^–^33^

Generally, common fluorescent dyes mainly rely on π–π* transition upon excitation, and they emit highly efficient fluorescence from their locally excited (LE) states.^34^ However, spin flip does not exist in OLEDs with LE-based emitters due to strong hole–electron pairs with a high exciton binding energy (∼1 eV),^35^ causing relatively low electron-to-photon conversion efficiency. On the other hand, charge transfer (CT) excitons, another type of excited species with loosely bound nature (∼10 meV) and typically found in D–A based molecular structures,^35^ can facilitate spin flip upon electrical charge injection and promote the exciton utilization efficiency (*η*_exc_) significantly.^36^ Nevertheless, the CT emitters with strong D–A structures often show red-shifted emissions accompanied with broad full-widths at half-maximum (FWHM), which is detrimental for the deep-blue color purity.^23^ Furthermore, low *Φ*_f_ is another drawback because of the lack of overlap between the frontier molecular orbitals.^37^ High values of *Φ*_f_ and *η*_exc_ are two prerequisites for obtaining a decent EQE, but the LE and the CT emitters fail to meet these requirements separately. To take full advantage of the LE and the CT excitons simultaneously, Ma and colleagues have established a series of highly emissive D–A type fluorescent materials that can employ more than 25% *η*_exc_ without using long-lived excitons.^26^,^36^,^38^ It is reported that moderate D–A pairs can lead to a hybrid local and charge transfer (HLCT) excited state, which is responsible for achieving high *Φ*_f_ and large *η*_exc_. In this excited system, the loosely bound excitons from the upper CT-like triplet state can be converted to singlets *via* a “hot exciton” channel, then produce photons from an LE-like singlet state.^39^ With these advantages, it is highly promising to design deep-blue D–A fluorescent materials employing the concept of HLCT excited states. However, until now OLEDs using emitters featuring HLCT excited states and having CIE_*y*_ coordinates below 0.06 and EQEs over 5% are still rarely reported.

**Scheme 1:**
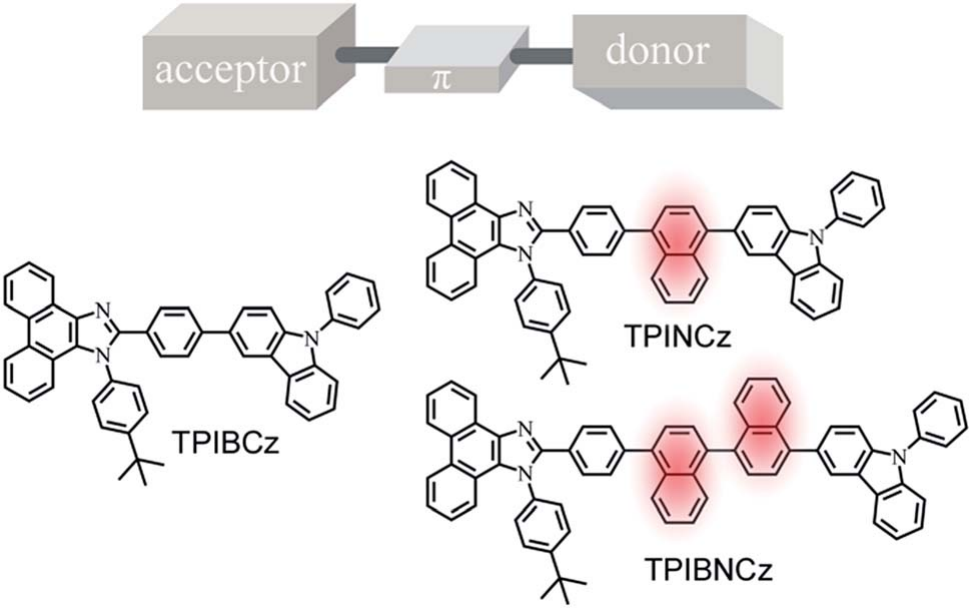
Chemical structures of the target D–π–A based fluorophores.

In this work, we report three D–π–A based PI–carbazole hybrid fluorophores—TPIBCz, TPINCz and TPIBNCz—with different π-linking moieties and apply them as the emitting cores in violet-blue OLEDs (Scheme 1). To obtain HLCT-based short-wavelength emitting molecules, D and A have to be carefully selected to obtain an appropriate electron push–pull strength such that both LE and CT excited states can be simultaneously achieved. In this molecular design, PI is chosen as a mild electron withdrawing group, while *N*-phenyl-9*H*-carbazole serves as a moderate electron donating segment. The PI and carbazole are both rigid planar groups, which are beneficial to achieving high *Φ*_f_. We also systematically studied the influence of π spacers, with different lengths and sizes between the D and the A moieties, on their photophysical properties and EL performances. All the materials efficiently emit violet-blue light and show excellent EL performance. It is worth noting that the OLED based on TPINCz as the emissive dopant exhibits a violet-blue color index of (0.153, 0.059) and a high efficiency with EQE up to 6.96 ± 0.08%. In a practical luminance of 1000 cd m^–2^, the EQE is still up to 6.56 ± 0.11%, which is the highest value among blue OLEDs with CIE_*y*_ coordinates below 0.08.

## Results and discussion

### Synthesis and characterization

The synthesis of the three new fluorescent materials is shown in Scheme S1 in the ESI.† First, the key intermediates AdhN-Br and AdhBN-Br were prepared by asymmetrical Suzuki reactions between the dibromo-naphthyl starting materials and (4-formylphenyl)boronic acid under mild conditions.^40^ Then the bromated PI derivatives TPI-Br, TPIN-Br and TPIBN-Br were synthesized *via* “one-pot” reactions.^41^ A mixture of an aromatic aldehyde (4-bromobenzaldehye, AdhN-Br or AdhBN-Br), phenanthrene-9,10-dione, 4-(*tert*-butyl)aniline, and ammonium acetate was reacted in refluxing CH_3_COOH under Ar for 10 h. At last, the target compounds were obtained by Suzuki reactions from bromated PI intermediates (TPI-Br, TPIN-Br and TPIBN-Br) and (*N*-phenyl-9*H*-carbazol-3-yl)boronic acid in good yields. The new compounds were characterized and determined by ^1^H/^13^C NMR and mass spectroscopy.

### Thermal properties

**Fig. 1:**
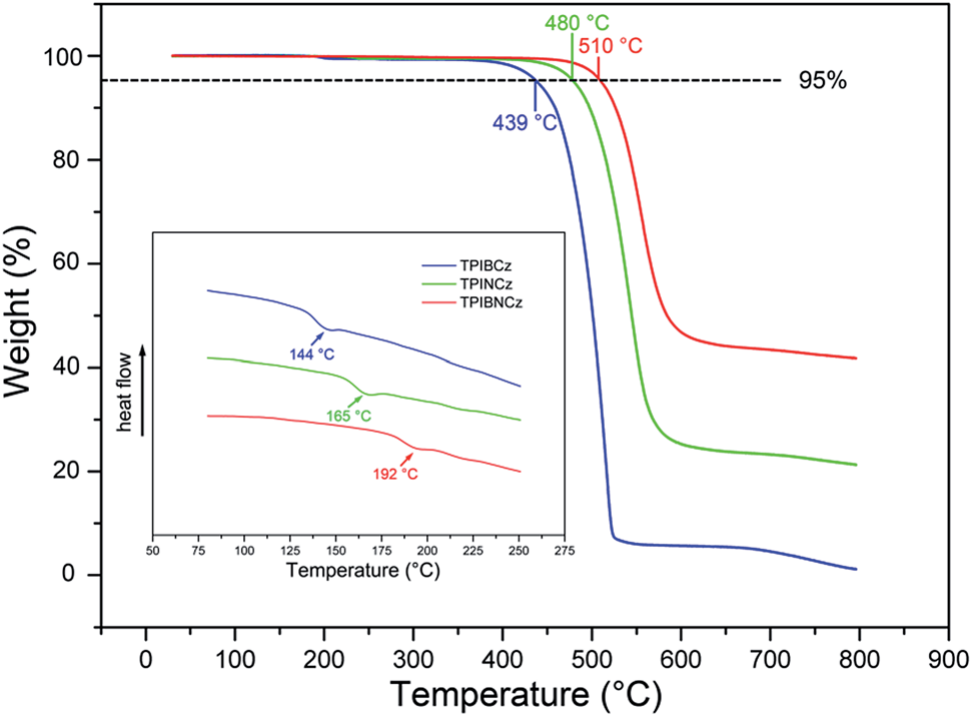
Thermal properties of the new materials.

The thermal properties of the new materials were characterized by thermogravimetric analysis (TGA) and differential scanning calorimetry (DSC) in a nitrogen atmosphere. The relevant data are listed in Table 1. Joule heat, which results from non-radiative energy loss and carrier injection among interfacial heterojunctions, is common in operating devices.^42^ Thus good thermal stability is of high significance for an OLED. The bulky and rigid structures of the new compounds are derived from the bulky conjugated backbones of PI and carbazole. The naphthyl-based compounds are more heat-resistant, with higher *T*_d_s (5% weight loss temperatures) of 480 and 510 °C for TPINCz and TPIBNCz, respectively (Fig. 1). Moreover, TPINCz and TPIBNCz have high ash values upon heating to 800 °C, nevertheless TPIBCz experiences complete weight loss, demonstrating that adoption of the naphthyl group is good for thermal stability.^43^ The high glass transition temperatures (*T*_g_s, inset of Fig. 1) also indicate the superior thermal stability of the as-designed materials, which are competent for general device fabrication and operation.

**Table 1:** Physical properties of TPIBCz, TPINCz and TPIBNCz

Compd	*T*_d_ ^*a*^ (°C)	*T*_g_ ^*b*^ (°C)	*E*_g_ ^*c*^ (eV)	HOMO^*d*^ (eV)	LUMO^*e*^ (eV)	*λ*_abs_ ^*f*^ (nm)	*λ*_em_ ^*f*^ (nm)	*Φ*_f_ ^*f*^ (%)
TPIBCz	439	144	3.04	–5.33	–2.29	338, 367/344, 370	395, 417, 428/414	85.2/64.2
TPINCz	480	165	3.06	–5.50	–2.44	335, 364/344, 369	428/450	∼100/90.5
TPIBNCz	510	192	3.14	–5.54	–2.40	334, 364/336, 368	425/433	∼100/96.8

^*a*^Decomposition temperature (5% weight loss).

^*b*^Glass transition temperature.

^*c*^Optical energy gap estimated from absorption onset in solid film.

^*d*^Measured by cyclic voltammetry.

^*e*^Calculated from LUMO = HOMO + *E*_g_.

^*f*^Measured in THF solution (10^–6^ mol L^–1^) and solid film (30 nm), respectively.

### Theoretical calculations

Theoretical calculations using the density functional theory (DFT) method on the B3LYP/6-31g(d,p) level are performed to study the structure–property relationship of the new compounds. All three compounds show similar molecular configurations apart from the central π-conjugated segments (Fig. S1†). Due to the naphthyl group providing a large steric hindrance, TPINCz and TPIBNCz have more twisted configurations than TPIBCz.^43^ This indicates that TPINCz and TPIBNCz can generate comparable emission to TPIBCz, although adoption of naphthyl group seems to extend the whole molecular conjugation to some degree.

**Fig. 2:**
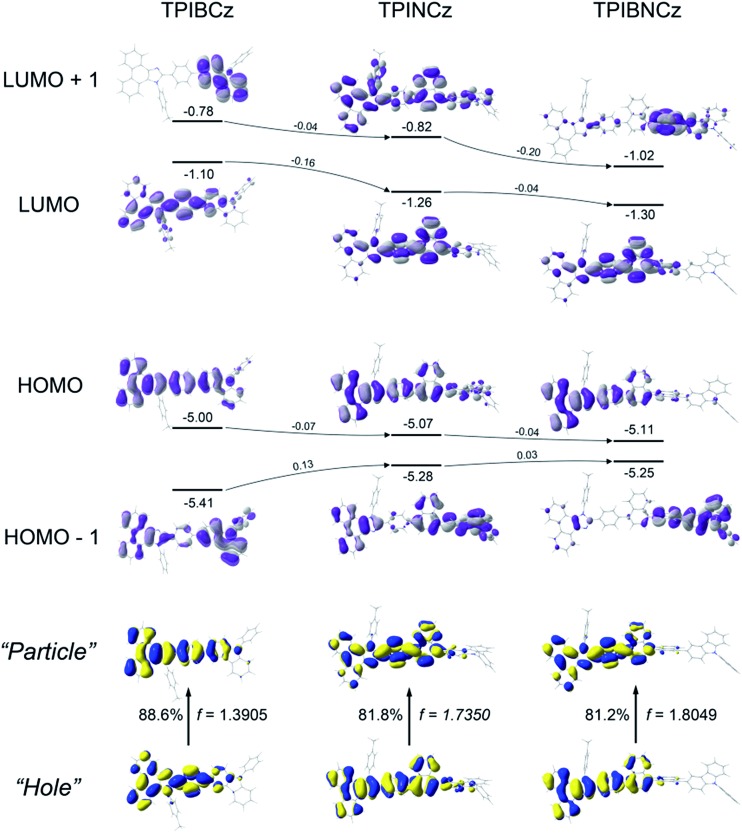
Spatial distributions of the molecular orbitals and S_0_ → S_1_ natural transition orbitals.

Fig. 2 shows the spatial distributions of the molecular orbitals of the new compounds. Except for TPIBNCz, the HOMOs of the other two molecules delocalize over the whole molecular framework with little contribution from the 4-(*tert*-butyl)phenyl group. The HOMO energy levels of the three compounds have slight differences, while those of HOMO–1 gradually shift from –5.41 eV of TPIBCz to –5.25 eV of TPIBNCz. In addition, we note that the energy offsets between the HOMO and the HOMO–1 energy levels become narrower from TPIBCz to TPIBNCz, leading to a pseudo-degenerate orbital pair in TPIBNCz. On the other hand, because of the incorporation of the naphthyl group with relatively large electron affinity,^44^ the energy levels of the LUMO and the LUMO+1 are deepened progressively, which is good for electron injection. Differences are also reflected in the orbital distribution, and the naphthyl/binaphthyl group dominates the electron density of the LUMOs in TPINCz and TPIBNCz. The naphthyl/binaphthyl group acts as a weak n-type π-linker in the D–π–A molecular design, and it plays a key role in manipulating the photophysical properties, which will be discussed later. Unlike the completely separated HOMO/LUMO distributions in the strong D–A based molecules, the relatively weak electron pushing and pulling strengths of the D and the A lead to partial overlap of the HOMOs/LUMOs in the present new compounds. TPINCz and TPIBNCz display more CT-like properties compared with TPIBCz due to the weak electron-withdrawing naphthyl group.

Natural transition orbital (NTO)^45^ analysis was implemented by time-dependent DFT (TD-DFT) calculations (based on TD-M062X/6-31G(d,p)) to further investigate the excited state properties. As shown in Fig. 2, both “hole” and “particle” are distributed on the PI and the central π-linking moieties for S_0_ (singlet ground state) → S_1_ (first singlet excited state) transitions in the three molecules, with little contribution from the *N*-phenyl-9*H*-carbazole. This indicates that PI may be an active emitting core, and the central π-linking pattern can influence the photophysical properties significantly. For TPIBCz, the “hole” and the “particle” seem to be located on the same part of the molecule, but the π spacer contributes more to the “hole” distribution. In comparison, the “hole” and the “particle” distributions have a certain superposition in the moiety from PI to naphthalene, but there is separation in the phenyl group between PI and naphthalene in TPINCz and TPIBNCz. The NTO analysis indicates that the three molecules may have LE character and show partial CT features for S_0_ → S_1_ transition. In addition, the introduction of naphthalene also enhances the oscillator strength (*f*), which helps to maintain high *Φ*_f_s.^46^

### Photophysical properties

**Fig. 3:**
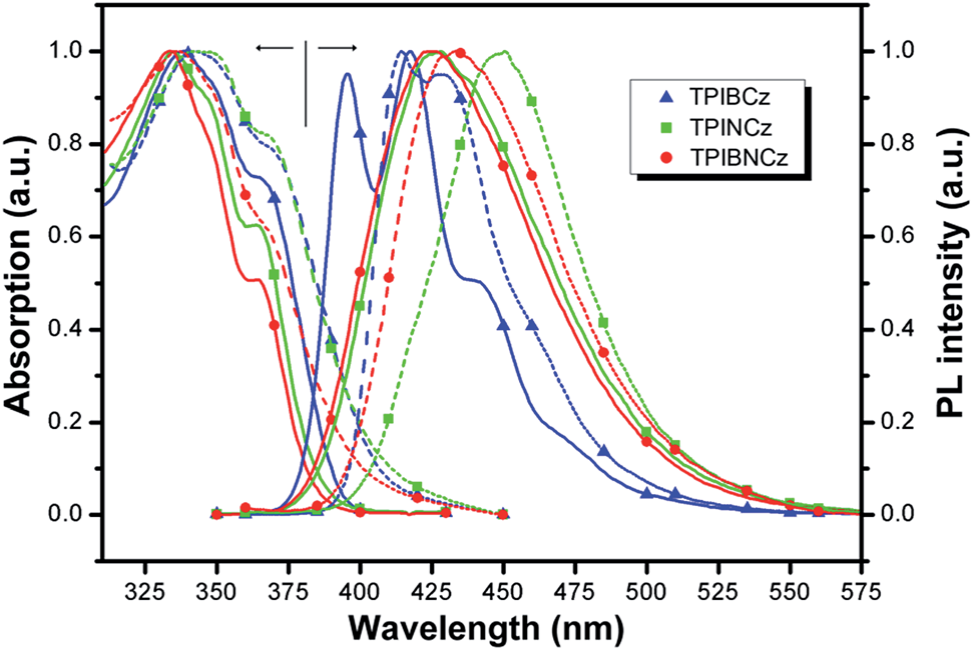
Absorption and PL spectra of TPIBCz, TPINCz and TPIBNCz; the solid lines and dashed lines are for the THF solution (∼10^–6^ mol L^–1^) and vacuum-evaporated film (30 nm), respectively.

The UV-vis absorption and photoluminescence (PL) spectra of the new compounds are shown in Fig. 3. The relative data are summarized in Table 1. In diluted THF solution, TPIBCz, TPINCz and TPIBNCz exhibit similar absorption bands, where the peaks between 330 and 340 nm are ascribed to the π–π* transition of the 2-substituent of imidazole to the PI group, while the sub-bands at the longer wavelength region (∼365 nm) are from PI’s π–π* transition.^47^ There are no evident broad absorptions, implying that the S_0_s are of negligible CT features. The absorptions in the solid film on quartz show slightly red-shifted spectra with respect to those in solutions. From the absorption onset, the optical energy gaps (*E*_g_s) are estimated to be 3.04, 3.06 and 3.14 eV, for TPIBCz, TPINCz and TPIBNCz, respectively. More twisted configurations induced by the naphthyl spacers may be responsible for the wider *E*_g_s in TPINCz and TPIBNCz. Combined with the results from cyclic voltammetry (Fig. S2†), in which the HOMO levels are measured to be –5.33, –5.50 and –5.54 eV, the LUMO levels are calculated to be –2.29, –2.44 and –2.40 eV for TPIBCz, TPINCz and TPIBNCz, respectively. All the materials can emit intense violet-to-blue fluorescence in diluted THF solution, but show quite different PL spectra. TPIBCz displays the highest energy emission in the violet-blue region with fine structural peaks maximized at 417 nm, which is similar to those observed in isolated phenyl-substituted PI.^48^,^49^ The relative *Φ*_f_ was measured to be 85.2%. Conversely, adding a naphthyl or binaphthyl to the π spacer results in structureless, red-shifted and relatively broad emission bands stemmed from the effects of conjugated extension and improved CT processes in transition. The fluorescence of TPINCz and TPIBNCz is very efficient with *Φ*_f_s approaching unity in the THF solution. Interestingly, TPIBNCz with a longer conjugated dimension has a shorter-wavelength emission than TPINCz. This indicates that the effect of the conjugated confinement induced by the large dihedral angle in the two-fold naphthyl spacer dominates the influence of the conjugated extension after inserting another naphthyl. This nicely meets the demand of molecular design for violet-blue emitters. In vacuum-evaporated solid films prepared on clean quartz, although slight red shifts in the emission are observed, the influence of close packing on emission is not significant, as evidenced by the high *Φ*_f_s (see Table 1) and relatively narrow emission bands, which are different from the typical excimer’s PL spectra.^50^ TPIBCz’s film also exhibits LE-like vibrational fine emission bands with a main peak at 414 nm. TPINCz shows the largest red shift in emission (peak at 450 nm), which results from polarization effect in the solid.^51^ This implies that the excited state of TPINCz is more sensitive to the polarity of the medium than that of TPIBCz.

**Fig. 4:**
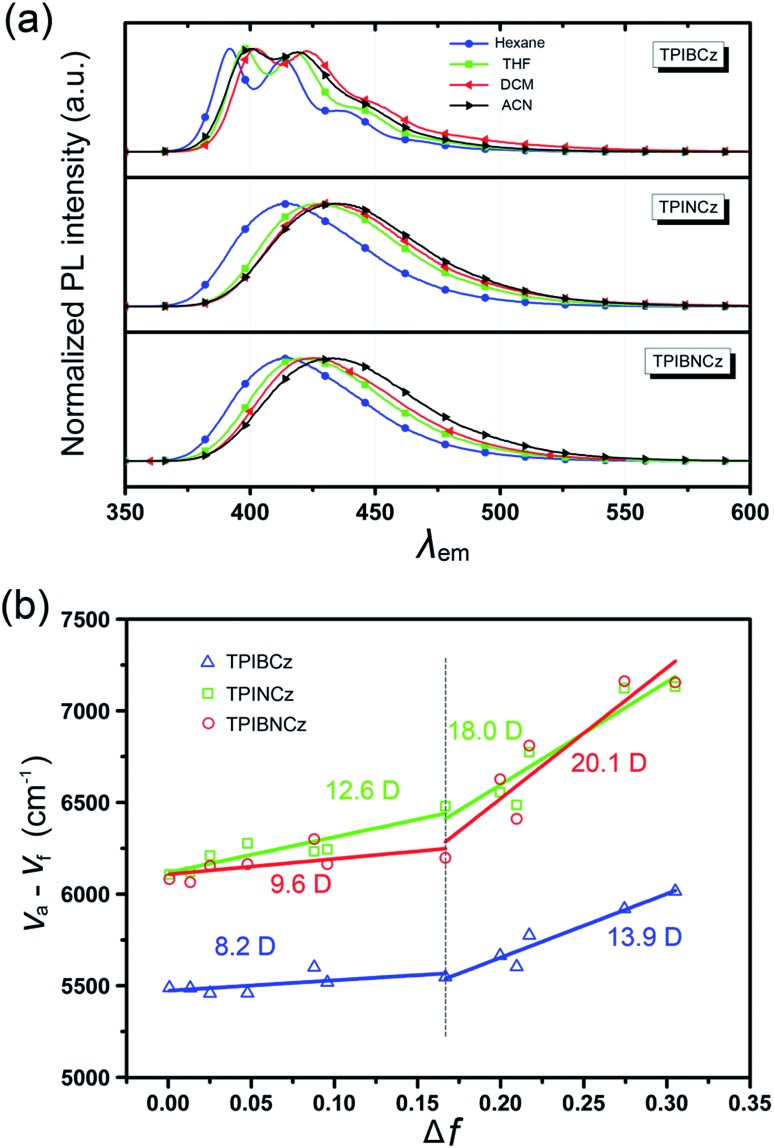
(a) Solvatochromic PL spectra of TPIBCz, TPINCz and TPIBNCz in solvents with increasing polarities; (b) linear fitting based on the Lippert–Mataga model in various solvents.

To further study the excited state properties, the solvation effects on the photophysical properties were investigated in solvents with different polarities (Fig. 4a and S3–S5†). Upon increasing the solvent polarities gradually from *n*-hexane to acetonitrile (ACN), TPIBCz constantly displays a sharp emission band and exhibits a slight red shift of 8 nm, which is responsible for the polarity-insensitive LE transition. For TPINCz and TPIBNCz, on the contrary, their PL spectra not only exhibit significant red shifts, but also progressively broadened structureless bands as the orientation polarization (Δ*f*) of the solvent increases. Compared with TPIBCz, such solvatochromic PL behavior in TPINCz and TPIBNCz implies CT properties in the excited states.^52^ The distinction of the solvatochromic effect in TPIBCz and TPINCz/TPIBNCz is also in accord with the calculated results’ hypothesis that the different properties of the excited state are induced by the naphthyl spacers. Based on the Lippert–Mataga model,^53^,^54^ we further estimated the dipole moments of the excited state (*μ*_e_) from the linear fitting analysis of the Stokes shift (*v*_a_ – *v*_f_) and Δ*f*. As shown in Fig. 4b, all three materials display two-section linear relations in the low and high Δ*f* regions, but the slopes of individual fittings are different. The two slopes for TPIBCz’s fittings are estimated to be 8.2 and 13.9 D, for the low and high Δ*f* regions, respectively. Based on the small *μ*_e_s, TPIBCz shows a LE excited state in the region of small Δ*f* and a LE-dominating HLCT excited state in the region of large Δ*f*. In contrast, although relatively small *μ*_e_s are obtained in TPINCz and TPIBNCz (12.6 and 9.6 D, respectively) in solvents with small Δ*f* values, the steep slopes are evident in solvents of high Δ*f* values, corresponding to *μ*_e_s of 18.0 and 20.1 D. The large *μ*_e_s can be treated as CT-like character. TPINCz and TPIBNCz show a mixture of LE and CT excited properties, namely the HLCT excited states. On the other hand, the two fitted lines for TPINCz show a smaller difference in their slopes compared to those for TPIBNCz. With this observation, we speculate that LE and CT excited states mix better in the former. In previous publications, the efficient HLCT excited states in blue emitters are generally achieved by connecting different donor and acceptor moieties with a single bond,^55^ different π-linking modes, or tuning the strength of electron donating/withdrawing in D/A pairs.^26^ In contrast, we can easily obtain accessible deep-blue emitters featuring HLCT excited states by incorporation of a naphthyl/binaphthyl spacer with weak n-type nature and high twisting angles into the π linking moiety, and this strategy can be applied to modify the common D–π–A to invoke the HLCT excited state.

### Electrical properties

The electrical properties of the three fluorophores were studied in single carrier-only type devices. The structure of the hole-only devices is indium tin oxide (ITO)/*N*,*N*′-bis-(1-naphthalenyl)-*N*,*N*′-bis-phenyl-(1,1′-biphenyl)-4,4′-diamine (NPB, 10 nm)/one of the new compounds (70 nm)/NPB (10 nm)/Al (150 nm), and the electron-only devices have a structure of ITO/2,9-dimethyl-4,7-diphenyl-1,10-phenanthroline (BCP, 10 nm)/one of the new compounds (70 nm)/BCP (10 nm)/LiF (1 nm)/Al (150 nm). The current density–voltage (*J*–*V*) curves are shown in Fig. S6.† Obviously, similar to most of the common organic semiconductors, the compounds have much better conductivity for hole than for electron. The relatively low hole transporting property of TPIBNCz may be caused by the highly twisting molecular configuration, as such geometry is not beneficial to charge flowing. On the other hand, the planar molecule TPIBCz has the highest electron mobility. The TPINCz-based electron-only device has lower current density than its TPIBNCz counterpart. This can be attributed to the incorporation of the binaphthyl spacer, which can enhance the LUMO overlap in solid state, although the highly twisting geometry more or less hinders charge transport.^43^

### Electroluminescence performance

**Fig. 5:**
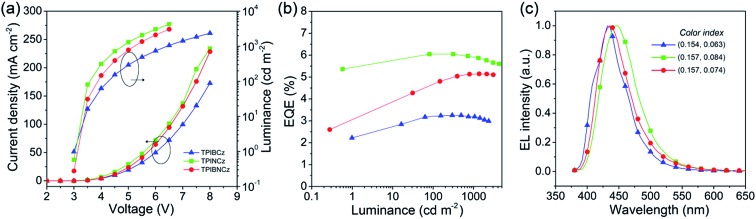
(a) Current density–voltage–luminance characteristics, (b) EQE–luminance curves, and (c) EL spectra of the TPIBCz, the TPINCz and the TPIBNCz based non-doped OLEDs.

To evaluate the EL performance of the new materials as emitting cores, we initially fabricated three non-doped OLEDs with a structure of ITO/NPB (70 nm for the TPIBCz and the TPIBNCz based devices, 50 nm for the TPINCz based OLED)/TcTa (tris(4-carbazoyl-9-ylphenyl)amine, 5 nm)/one of the emitters (30 nm)/1,3,5-tris(1-phenyl-1*H*-benzimidazol-2-yl)benzene (TPBi, 30 nm)/LiF (1 nm)/Al (100 nm), in which ITO is the transparent anode, NPB is used as a hole transporting layer, TcTa as a buffer and exciton confining layer,^24^ TPBi as an electron transporting and hole blocking layer, LiF as an electron injection layer and Al is the cathode. Although the emitters used here have better hole than electron transporting properties, the unfavorable effects due to the unbalanced electron and hole currents are partly remedied by the exciton confining structures. The thin TcTa layer can hinder the hole flow in the device due to the additional interfacial heterojunction, and it can confine excitons in the emissive layer due to its high *E*_g_ (∼3.4 eV). Besides, the deep HOMO level (–6.30 eV) of TPBi can also block the hole in the devices. Fig. 5 shows the device performances, and the key performance data are summarized in Table 2. The OLED using TPINCz displays the highest EQE of 5.95 ± 0.10% and deep-blue EL emission with CIE coordinates of (0.157, 0.084) (at 1000 cd m^–2^). It is worth noting that this device does not show serious efficiency roll-off. At a practical luminance of 1000 cd m^–2^, a high-level EQE of 5.83 ± 0.06% is still maintained. The maximum current efficiency (CE) and PE are up to 5.00 ± 0.14 cd A^–1^ and 5.15 ± 0.15 lm W^–1^, respectively. The devices based on TPIBCz and TPIBNCz show slightly lower performances and have maximum EQEs of 3.38 ± 0.10% and 5.09 ± 0.13%, respectively. But their EL spectra shift to the shorter-wavelength region with peaks at 435 and 436 nm, and the corresponding CIE coordinates are (0.154, 0.063) and (0.157, 0.074) at 1000 cd m^–2^, respectively. Fig S7† shows the EL and PL spectra of the non-doped devices and solid thin films. The two types of spectra have a slight difference in bandwidth which might be due to weak micro-cavity effects in the devices. The EL spectra are comparable to the solid film PL spectra, which indicates that the devices emit intrinsic emission from the titular fluorophores. It is suggested that the highest performances attained in TPINCz-based OLEDs could be ascribed to the high CT component in the excited state and better hybridization of the LE and the CT states. Fig. S8† shows the calculated first-ten singlet and triplet energy levels of the three molecules. Some upper triplet states are close to their corresponding S_1_s, like T_5_/T_6_, T_7_ and T_9_ for TPIBCz, TPINCz and TPIBNCz, respectively. In addition, the NTOs of these high-lying triplet states exhibit complicated transitions with similar contributions and HLCT excited properties (Fig. S9–S11†). This implies that this high performance may be due to the potential “hot exciton” channels through reverse inter-system crossing (RISC) from upper triplet states to S_1_ (red arrows in Fig. S8†). Contributions to these high efficiencies from TF and TADF are considered to be insignificant. The linear relation of the luminance–current density (*L*–*J*) curves (Fig. S12†) implies that there are few emissive triplet excitons produced *via* TF,^56^ and there are large S_1_ and T_1_ energy gaps (Fig. S8†) as well as certain HOMOs/LUMOs overlap (Fig. 2) indicating that the new molecules may not emit TADF.^7^

**Table 2:** EL performances of the OLEDs based on TPIBCz, TPINCz and TPIBNCz

Emissive layer	*V*_on_ ^*a*^ (V)	*λ*_EL_ (nm)	CIE^*b*^ (*x*, *y*)	CE_max_^*c*^ (cd A^–1^)	PE_max_^*d*^ (lm W^–1^)	EQE_max_^*e*^ (%)
TPIBCz	3.0	435	0.154, 0.063	1.70 ± 0.02	1.44 ± 0.05	3.38 ± 0.10
TPINCz	3.1	448	0.157, 0.084	5.00 ± 0.14	5.15 ± 0.15	5.95 ± 0.10
TPIBNCz	3.2	436	0.157, 0.074	3.29 ± 0.08	2.80 ± 0.09	5.09 ± 0.13
10 wt% TPIBCz	3.7	428	0.156, 0.043	1.91 ± 0.02	1.72 ± 0.02	5.06 ± 0.05
20 wt% TPIBCz	3.3	432	0.156, 0.046	1.96 ± 0.02	1.68 ± 0.04	5.46 ± 0.07
30 wt% TPIBCz	3.2	432	0.156, 0.047	2.10 ± 0.04	2.20 ± 0.08	5.47 ± 0.10
10 wt% TPINCz	3.3	436	0.155, 0.054	3.02 ± 0.15	2.53 ± 0.20	6.60 ± 0.20
20 wt% TPINCz	3.1	440	0.153, 0.059	3.71 ± 0.11	3.71 ± 0.15	6.96 ± 0.08
30 wt% TPINCz	3.1	440	0.153, 0.062	3.75 ± 0.06	3.36 ± 0.05	6.71 ± 0.12
10 wt% TPIBNCz	3.4	424	0.158, 0.044	1.89 ± 0.05	1.51 ± 0.10	5.48 ± 0.15
20 wt% TPIBNCz	3.2	428	0.157, 0.048	2.34 ± 0.04	2.14 ± 0.04	5.99 ± 0.05
30 wt% TPIBNCz	3.1	428	0.157, 0.051	2.55 ± 0.05	2.24 ± 0.10	6.03 ± 0.14

^*a*^Voltage at 1 cd m^–2^.

^*b*^Detected at 1000 cd m^–2^.

^*c*^Current efficiency.

^*d*^Power efficiency.

^*e*^External quantum efficiency at maximum.

**Fig. 6:**
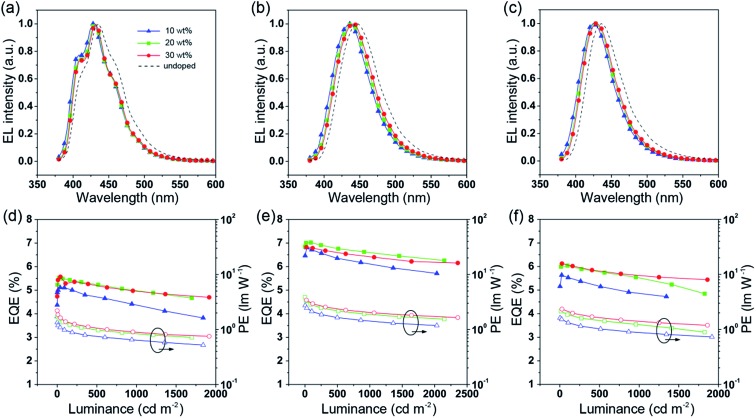
EL spectra of the CBP-doped devices based on (a) TPIBCz, (b) TPINCz and (c) TPIBNCz. The EQE–luminance curves for the CBP-doped devices based on (d) TPIBCz, (e) TPINCz and (f) TPIBNCz.

**Fig. 7:**
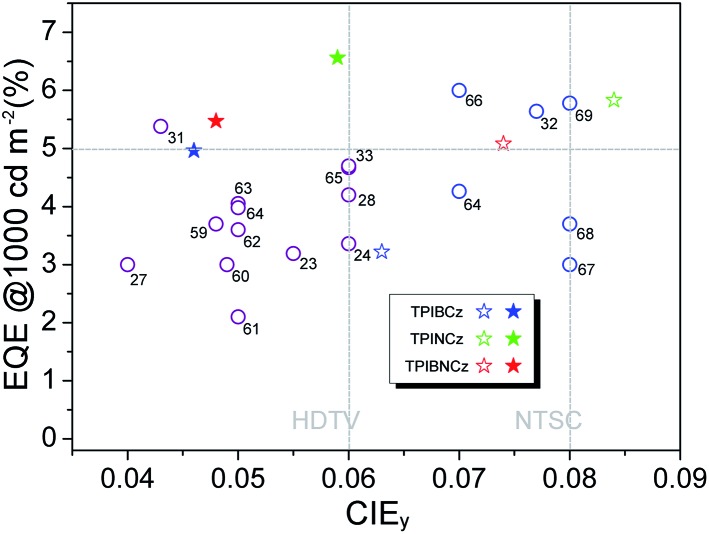
A plot of EQE at 1000 cd m^–2^ against CIE_*y*_ for the state-of-the-art deep-blue OLEDs with CIE_*y*_ ≤0.08. The data points marked with empty circles and the reference numbers are taken from published devices. The details of these devices are listed in the ESI (Table S2†). The data points marked with empty and solid stars are devices developed in this work by using the three emitters in the non-doped and doped emitting layers, respectively.

To further improve the efficiency and color purity, we also fabricated doped devices with a structure of ITO/NPB (70 nm)/TcTa (5 nm)/CBP (4,4′-bis(*N*-carbazolyl)-1,1′-biphenyl) + one of the emitters (30 nm)/TPBi (30 nm)/LiF (1 nm)/Al (100 nm). The influence of different doping levels (10, 20 and 30 wt%) on the device performances was investigated. CPB, a high-energy-gap compound, was chosen as a host material. In the host–guest system, the efficiency can be improved significantly due to efficient Förster energy transfer.^57^ Besides, in the host matrix, better color purity, wider recombination zone and less non-radiative relaxation can be expected. Fig. 6a–c show the EL spectra of the devices. All the doped devices show obviously blue-shifted EL spectra compared with the corresponding non-doped devices. This can be mainly attributed to the decrease in the medium polarity, since CBP is a non-polar host material.^58^ As the dopant concentration decreases, the finer structural EL spectra are observed in the TPIBCz-based devices, which is in accord with the LE-like photophysical behavior of TPIBCz. The corresponding CIE_*y*_ coordinate shifts from 0.063 (non-doped) to 0.043 (10 wt% in CBP). On the other hand, the EL spectra of the TPINCz and the TPIBNCz-based OLEDs are relatively more sensitive to doping ratio, which may be responsible for the CT excited properties. For example, the EL spectra of the TPINCz-based doped devices show an evidently hypsochromic shift from the deep-blue region (*λ*_EL_ = 448 nm, CIE: (0.152, 0.084)) in the non-doped device to the violet-blue region (*λ*_EL_ = 436 nm, CIE: (0.155, 0.054)). Similar changes are observed in the TPIBNCz-based OLEDs. The doped devices show superior violet-blue EL emission, their corresponding CIE_*y*_ coordinates range from ∼0.04 to 0.06, even surpassing the standard blue for the National Television System Committee (NTSC) of (0.14, 0.08) and the HDTV of (0.15, 0.06), as shown in Fig. S13.† Fig. 6d–f show the EQE–*L*–PE curves for the doped devices, and their *J*–*V*–*L* characteristics are shown in Fig. S14.† The doped OLEDs show improved efficiency. The maximum EQE of the TPIBCz-based 10 wt% doped OLED reaches 5.06 ± 0.05% compared to the value of 3.38 ± 0.10% in the non-doped device. However, the *V*_on_ is raised to 3.7 V, mainly due to the larger energy gap of CBP. Upon increasing the dopant concentration from 10 to 30 wt%, the device efficiency first increases and then nearly saturates at 30 wt%. The electrical property also follows this trend. The current density is the highest and the *V*_on_ decreases to 3.2 V in the 30 wt% TPIBCz-based device. Similar changes are observed in the TPINCz and the TPIBNCz-based OLEDs. However, the EL spectra gradually shift to the longer-wavelength region as the dopant concentration increases. Comprehensively speaking, we suggest that the optimized doping level is 20 wt%. Among all of the devices fabricated, the TPINCz-based doped device (20 wt%) exhibits the highest performance with an EQE up to 6.96 ± 0.08% and a decent maximum PE of 3.71 ± 0.15 lm W^–1^. The highest performance of the TPINCz-based device may be mainly ascribed to the better mixed LE and CT components, resulting in a more efficient HLCT excited state. The EL performances of the reported high-efficiency deep-blue OLEDs are summarized in Table S2,† and illustrated in Fig. 7.^23^,^24^,^27^,^28^,^31^–^33^,^59^–^69^ It can be seen that few OLEDs can emit efficient violet-blue light with EQE over 5% at a luminance of 1000 cd m^–2^. The OLEDs based on our new emitters exhibit superior efficiencies over most of the reported devices with CIE_*y*_ <0.08 (NTSC standard) at a luminance of 1000 cd m^–2^. More importantly, the TPINCz-based doped OLED (red solid star) shows a champion performance with an EQE of 6.56 ± 0.11% at 1000 cd m^–2^.

## Conclusions

We have designed and synthesized three D–π–A type PI–carbazole hybrid fluorophores: TPIBCz, TPINCz and TPIBNCz. It is found that the pattern of the π spacers has significant influence on the photophysical properties and device performances. The incorporation of a naphthyl/binaphthyl group between 1-(4-(*tert*-butyl)phenyl)-2-phenyl-1*H*-phenanthro[9,10-*d*]imidazole and *N*-phenyl-9*H*-carbazole can alleviate the common dilemma that enhancing device efficiency by increasing CT excited properties often leads to red-shifted emission. The quantum calculations and photophysical experiments demonstrated that the naphthyl-linked TPINCz and TPIBNCz show remarkable HLCT excited states, while TPIBCz exhibits a LE-dominating character. Our materials can emit violet-blue emission with high *Φ*_f_s in solid films, indicating that they are promising candidates for high-performance violet-blue emitters in OLED applications. The doped device of TPINCz shows violet-blue EL with CIE coordinates of (0.153, 0.059), which are very close to the HDTV blue color standard of (0.15, 0.06). Its maximum EQE can reach 6.96 ± 0.08%. Additionally, this value retains 94% of the peak value at a practical brightness of 1000 cd m^–2^, showing a very low efficiency roll-off. The performances of the OLEDs reported here are among the best for the devices with CIE_*y*_ ≤0.08. Our study may provide a new pathway for designing high-performance violet-blue emitters for OLED applications.

## Supplementary Material

Supplementary information
