# Supplementary material for: Achieving efficient violet-blue electroluminescence with CIEy <0.06 and EQE >6% from naphthyl-linked phenanthroimidazole–carbazole hybrid fluorophores
Source: Chem Sci. 2017 Feb 20;8(5):3599–608. doi: 10.1039/c6sc05619a (PMC6094158; doi:10.1039/c6sc05619a)
Supplement: Supplementary file 1 [file SC-008-C6SC05619A-s001.pdf]

Electronic Supplementary Material (ESI) for Chemical Science.
This journal is © The Royal Society of Chemistry 2017

Electronic Supplementary Information

Achieving Efficient Violet-Blue Electroluminescence with CIE_y < 0.06 and EQE > 6% from Naphthyl-Linked Phenanthroimidazole-Carbazole Hybrid Fluorophores

Wen-Cheng Chen,[a] Yi Yuan,[ab] Shao-Fei Ni,[c] Qing-Xiao Tong,*[b] Fu-Lung Wong,[a] Chun-Sing Lee*[a]

[a] Center of Super-Diamond and Advanced Films (COSDAF) and Department of Chemistry, City University of Hong Kong, Hong Kong SAR, PR China
E-mail: apcslee@cityu.edu.hk
[b] Department of Chemistry and Key Laboratory for Preparation and Application of Ordered Structural Materials of Guangdong Province, Shantou University, 243 University Road, Shantou, Guangdong, 515063, PR China
E-mail: qxtong@stu.edu.cn
[c] Department of Chemistry, Southern University of Science and Technology, Shenzhen, 518055, PR China

Contents

General information 1
Synthesis detail 1
Device fabrication and measurement. 4
Optimized molecular configuration 5
Cyclic voltammetry 5
Solvatochromic effects 6
Single carrier-only devices 7
EL versus PL spectra 8
TD-DFT calculation 8
Luminance-current density characteristics 12
CIE map 12
Current density-voltage-luminance characteristics 13
Performance comparison 14
References 15

General information

^{1}H and ^{13}C NMR spectra were obtained on a Varian Unity Inova 400 spectrometer. Mass spectra were measured via a Thermo ISQ mass spectrometer with a direct exposure probe. Decomposition temperatures were determined on a TA Instrument TGAQ50 at a heating rate of 10 °C minute^{-1} under N_2 protection, while a Perkin-Elmer DSC 7 differential scanning calorimetric was used to measure the glass transition temperatures. UV-vis absorption and photoluminescence spectra were scanned on a Perkin-Elmer Lambda 950 UV/vis spectrometer and a Perkin-Elmer LS50B spectrophotometer, respectively. Relative photoluminescence quantum yields in THF solution were estimated using 9,10-diphenylanthracene cyclohexane solution as the standard reference (90%).[1] Absolute photoluminescence quantum yields of the organic films were measured with a Labsphere™ integrating sphere. Cyclic voltammetry was detected on a CHI600 voltammetric analyzer equipped with a three-electrode system (platinum disk the working electrode, platinum wire the auxiliary electrode, Ag/AgCl the reference electrode). Fc/Fc^{+} with an absolute HOMO level of -4.80 eV was used as the internal standard. Nitrogen-saturated 0.1 mol L^{-1} tetrabutylammonium hexafluorophosphate dichloromethane solution was used as the supporting electrolyte. Chemicals and reagents were used directly from commercial suppliers without further purification.

Synthesis detail

The starting material 4,4'-dibromo-1,1'-binaphthalene[2] and intermediate TPI-Br[3] were prepared according to the literatures. Synthetic route of the title compounds is outlined in Scheme S1.

4-(4-bromonaphthalen-1-yl)benzaldehyde (AdhN-Br). The product was obtained by refluxing 1,4-dibromonaphthalene (1.43 g, 5 mmol), (4-formylphenyl)boronic acid (0.75 g, 5 mmol) and 0.29 g $Pd(PPh_3)_4$, (0.29 g, 0.25 mmol) in a mixture of 5 mL sat. Na_2CO_3 aq. and 40 mL THF for 5 h under Ar. The mixture was allowed to cool to room temperature and washed with deionized water before extracting with CH_2Cl_2. The organic layer was separated

and dried with anhydrous $MgSO_4$ and concentrated by rotary evaporation. At last the raw product was purified by flash column chromatography using mixture of petroleum ether and CH_2Cl_2 to obtained 0.96 g white powder (yield: 61.9%) ^{1}H NMR (400 MHz, $CDCl_3$) δ [ppm]: 10.13 (s, 1H), 8.36 (d, J = 8.5 Hz, 1H), 8.02 (d, J = 8.0 Hz, 2H), 7.87 (d, J = 7.6 Hz, 1H), 7.81 (d, J = 8.5 Hz, 1H), 7.64 (t, J = 6.6 Hz, 3H), 7.51 (t, J = 7.6 Hz, 1H), 7.31-7.27 (m, 1H). EI MS (m/z): 310.11.

2-(4-(4-bromonaphthalen-1-yl)phenyl)-1-(4-(*tert*-butyl)phenyl)-1*H*-phenanthro[9,10-*d*]imidazole (TPIN-Br). 0.93 g (3 mmol) AdhN-Br, 0.83 g (4 mmol) phenanthrene-9,10-dione, 0.60 g (4 mmol) 4-(*tert*-butyl)aniline and 2.31 g (30 mmol) $AcONH_4$ were added to a two-necked flask. 30 mL glacial acetic acid was added under stirring and then the mixture was refluxed under Ar for 10 h. After cooling to room temperature, 100 mL methanol was poured to the mixture and stirred for further 2 h. The resulting yellow-green suspension was then filtered and the residue was dried in a vacuum oven for 1 h, followed by purification by silica gel column chromatography (CH_2CH_2 as eluent) to yield an off-white powder (94.9%). ^{1}H NMR (400 MHz, $CDCl_3$) δ [ppm]: 8.91 (d, J = 7.9 Hz, 1H), 8.79 (d, J = 8.4 Hz, 1H), 8.73 (d, J = 8.2 Hz, 1H), 8.32 (d, J = 8.5 Hz, 1H), 7.94-7.57 (m, 10H), 7.57-7.44 (m, 4H), 7.39 (d, J = 8.2 Hz, 2H), 7.30 (d, J = 8.3 Hz, 2H), 1.46 (s, 9H). EI MS (m/z): 631.82.

4-(4'-bromo-[1,1'-binaphthalen]-4-yl)benzaldehyde (AdhBN-Br). The synthetic process is similar with AdhN-Br to yield a white powder (69.2%). ^{1}H NMR (400 MHz, $CDCl_3$) δ [ppm]: 10.16 (s, 1H), 8.38 (d, J = 8.5 Hz, 1H), 8.08 (d, J = 8.0 Hz, 2H), 7.94 (d, J = 7.7 Hz, 2H), 7.79 (d, J = 7.9 Hz, 2H), 7.62 (t, J = 7.6 Hz, 1H), 7.55 (s, 2H), 7.46 (t, J = 7.6 Hz, 3H), 7.39 (d, J = 7.6 Hz, 2H), 7.34 (dd, J = 12.7, 5.4 Hz, 1H). EI MS (m/z): 437.39.

2-(4-(4'-bromo-[1,1'-binaphthalen]-4-yl)phenyl)-1-(4-(*tert*-butyl)phenyl)-1*H*-phenanthro[9,10-*d*]imidazole (TPIBN-Br). The synthetic process is similar with TPIN-Br to yield a white powder (78.5%). ^{1}H NMR (400 MHz, $CDCl_3$) δ [ppm]: 8.93 (d, J = 7.8 Hz, 1H), 8.80 (d, J = 8.4 Hz, 1H), 8.74 (d, J = 8.3 Hz, 1H), 8.37 (d, J = 8.4 Hz, 1H), 7.98 (d, J = 9.0 Hz, 1H), 7.92 (d, J = 7.5 Hz, 1H), 7.82-7.73 (m, 3H), 7.72-7.27 (m, 19H), 1.47 (s, 9H). EI MS (m/z): 758.29.

1-(4-(*tert*-butyl)phenyl)-2-(4-(9-phenyl-9H-carbazol-3-yl)phenyl)-1*H*-phenanthro[9,10-*d*]imidazole (TPIBCz). A mixture of TPI-Br (1.01 g, 2 mmol) and (N-phenyl-9*H*-carbazol-3-

yl)boronic acid (0.69 g, 2.4 mmol) were dissolved in 60 mL toluene, and then 20 mL 2 M Na_2CO_3 aq., 10 mL ethanol and 0.12 g (0.1 mmol) $Pd(PPh_3)_4$ were added under stirring. The mixture was heated to 90 °C under Ar. After overnight reaction, the mixture was cool to room temperature and washed with deionized water and then extracted with chloroform. After separation, the organic layer was dried over anhydrous $MgSO_4$ and the solvent was removed under vacuum. Finally, the crude product was purified by column chromatography by using CH_2Cl_2 as eluent to yield 1.21 g white powder (yield: 90.7%). ^1H NMR (400 MHz, $CDCl_3$) δ [ppm]: 8.92 (d, J = 7.9 Hz, 1H), 8.78 (d, J = 8.4 Hz, 1H), 8.72 (d, J = 8.4 Hz, 1H), 8.36 (s, 1H), 8.19 (d, J = 7.7 Hz, 1H), 7.82-7.55 (m, 13H), 7.55-7.39 (m, 7H), 7.36-7.27 (m, 2H), 7.22 (d, J = 8.2 Hz, 1H), 1.47 (s, 9H). ^{13}C NMR (151 MHz, $CDCl_3$) δ [ppm]: 153.26, 150.82, 142.03, 141.35, 140.53, 137.55, 136.09, 132.44, 129.90, 129.67, 129.20, 128.57, 128.33, 128.22, 127.55, 127.21, 127.06, 127.04, 126.83, 126.18, 125.50, 125.27, 124.72, 124.03, 123.89, 123.38, 123.16, 123.06, 122.77, 120.89, 120.35, 120.11, 118.69, 110.04, 109.93, 35.04, 31.42. EI MS (m/z): 667.56.

1-(4-(*tert*-butyl)phenyl)-2-(4-(4-(9-phenyl-9*H*-carbazol-3-yl)naphthalen-1-yl)phenyl)-1*H*-phenanthro[9,10-*d*]imidazole (TPINCz). The synthetic process is similar with TPIBCz to yield an off-white powder (71.2%). ^1H NMR (400 MHz, $CDCl_3$) δ [ppm]: 8.93 (d, J = 7.8 Hz, 1H), 8.80 (d, J = 8.4 Hz, 1H), 8.74 (d, J = 8.4 Hz, 1H), 8.29 (s, 1H), 8.16 (d, J = 7.7 Hz, 1H), 8.12-8.04 (m, 1H), 8.01-7.93 (m, 1H), 7.77 (dd, J = 10.3, 8.0 Hz, 3H), 7.67 (dd, J = 12.2, 5.6 Hz, 7H), 7.62 -7.40 (m, 14H), 7.35-7.28 (m, 3H), 1.47 (s, 9H). ^{13}C NMR (151 MHz, $CDCl_3$) δ [ppm]: 153.33, 150.80, 141.32, 140.76, 140.24, 138.70, 137.67, 136.01, 132.49, 131.77, 129.99, 129.92, 129.25, 129.20, 128.60, 128.31, 128.26, 128.20, 127.54, 127.26, 127.10, 127.05, 126.82, 126.79, 126.42, 126.23, 126.14, 125.85, 125.55, 124.82, 124.06, 123.41, 123.30, 123.15, 123.08, 122.77, 121.72, 120.94, 120.36, 120.07, 109.92, 109.49, 35.05, 31.41. EI MS (m/z): 792.38.

1-(4-(*tert*-butyl)phenyl)-2-(4-(4'-(9-phenyl-9*H*-carbazol-3-yl)-[1,1'-binaphthalen]-4-yl)phenyl)-1*H*-phenanthro[9,10-*d*]imidazole (TPIBNCz). The synthetic process is similar with TPIBCz to yield a white powder (88.0%). ^1H NMR (400 MHz, $CDCl_3$) δ [ppm]: 8.94 (d, J = 7.4 Hz, 1H), 8.81 (d, J = 8.5 Hz, 1H), 8.75 (d, J = 8.2 Hz, 1H), 8.39 (s, 1H), 8.16 (dt, J = 13.7, 6.4 Hz, 2H), 8.01 (d, J = 8.4 Hz, 1H), 7.82-7.74 (m, 3H), 7.72-7.39 (m, 24H), 7.39-7.28

(m, 5H), 7.25 (s, 1H), 1.48 (d, J = 4.9 Hz, 9H). ^{13}C NMR (151 MHz, $CDCl_3$) δ [ppm]: 141.34, 140.86, 140.27, 137.69, 137.57, 133.26, 133.21, 132.58, 132.23, 131.48, 130.04, 129.93, 129.24, 128.59, 128.29, 127.55, 127.48, 127.45, 127.13, 127.07, 126.88, 126.66, 126.42, 126.25, 126.15, 125.97, 125.89, 125.81, 125.76, 124.07, 123.44, 123.35, 123.09, 121.83, 120.95, 120.37, 120.09, 109.93, 109.52, 35.06, 31.42, 26.89. EI MS (m/z): 919.43.

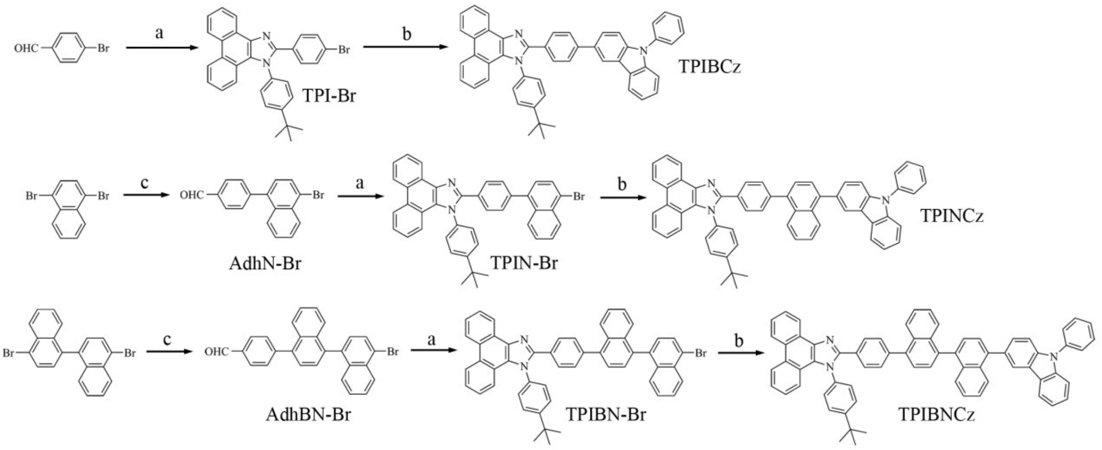

Scheme S1 Synthesis routes for TPIBCz, TPINCz and TPIBNCz. a: phenanthrene-9,10-dione, 4-(*tert*-butyl)aniline, $AcONH_4$, AcOH reflux under Ar; b: (N-phenyl-9*H*-carbazol-3-yl)boronic acid, $Pd(PPh_3)_4$, toluene/EtOH/Na_2CO_3 aq. (2 M), 90° C under Ar; c: (4-formylphenyl)boronic acid, $Pd(PPh_3)_4$, sat. Na_2CO_3 aq./THF reflux under Ar.

Device fabrication and measurement

Devices were fabricated on cleaned ITO-coated glass substrates having a sheet resistance of 15 Ω □$^{-1}$. Before use, ITO substrates were swabbed with Decon-90 aqueous solution, before 15-min ultrasonic baths in acetone and deionized water respectively, and then rinsed with isopropanol. Finally, N_2 flow was used to remove isopropanol on the surface, and then the substrates were dried in an oven at 120 °C. After a 15-minute UV-ozone cleaning, the ITO substrates were loaded into a deposition chamber with a base vacuum below 10^{-6} torr. Deposition rates were monitored with a quartz oscillating crystal. Current density-voltage characteristics and electroluminescence luminance were measured with a Keithley 237 power source and a Spectrascan PR650 photometer, respectively. Device measurements were carried out under ambient conditions without encapsulation.

Optimized molecular configuration

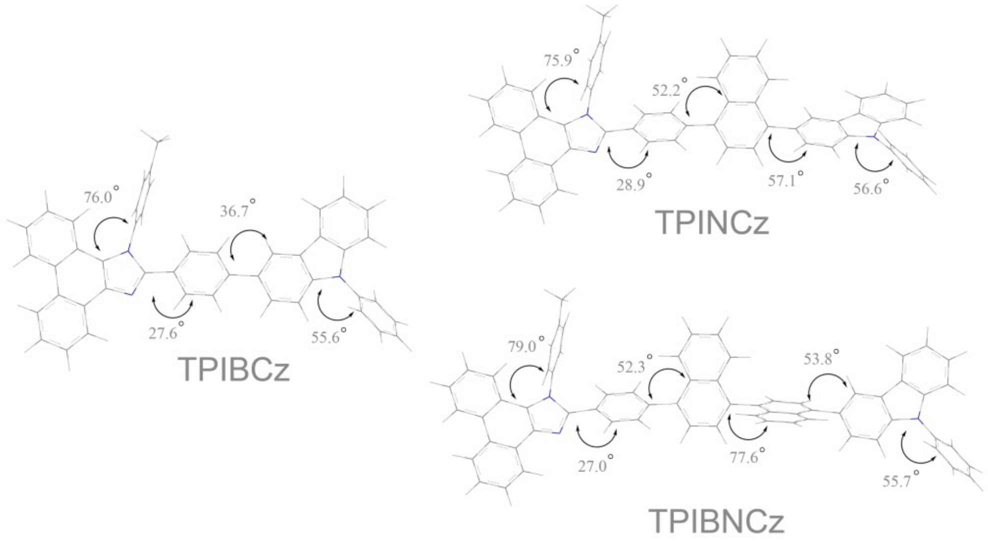

Fig. S1 Optimized molecular configurations of the new compounds.

Cyclic voltammetry

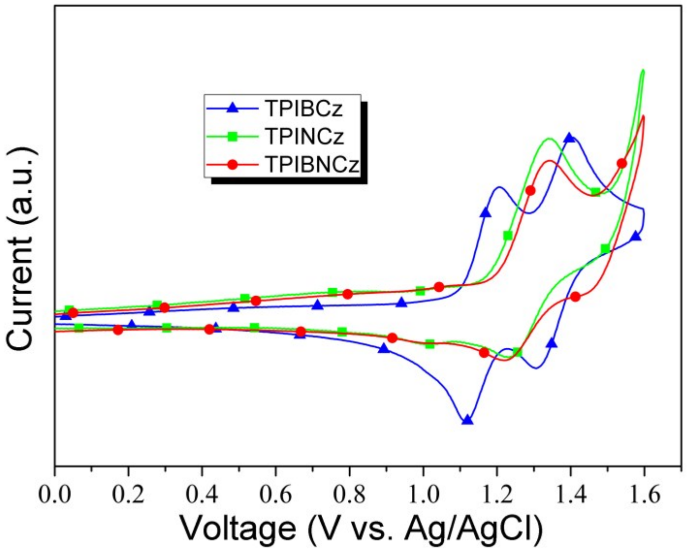

Fig. S2 Cyclic voltammogram for oxidation of TPIBCz, TPINCz and TPIBNCz.

Solvatochromic effects

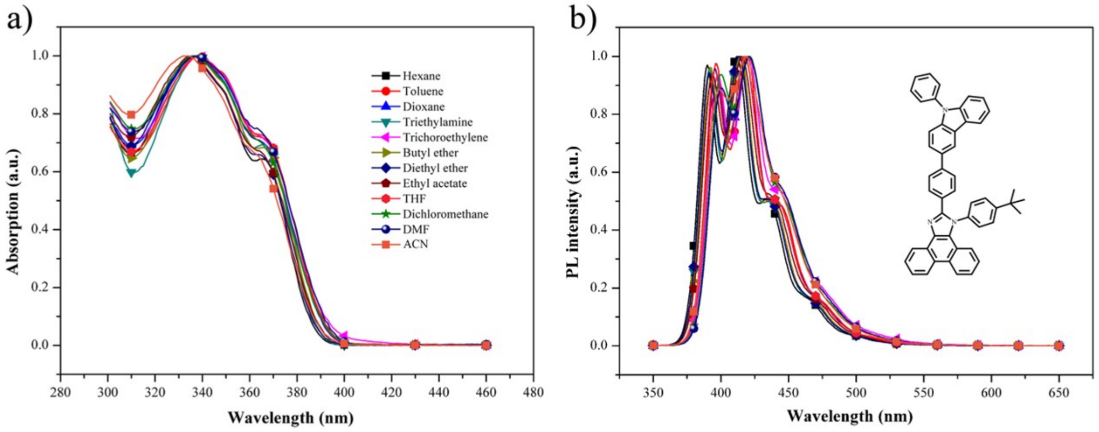

Fig. S3 Solvent-dependent absorption and PL spectra of TPIBCz.

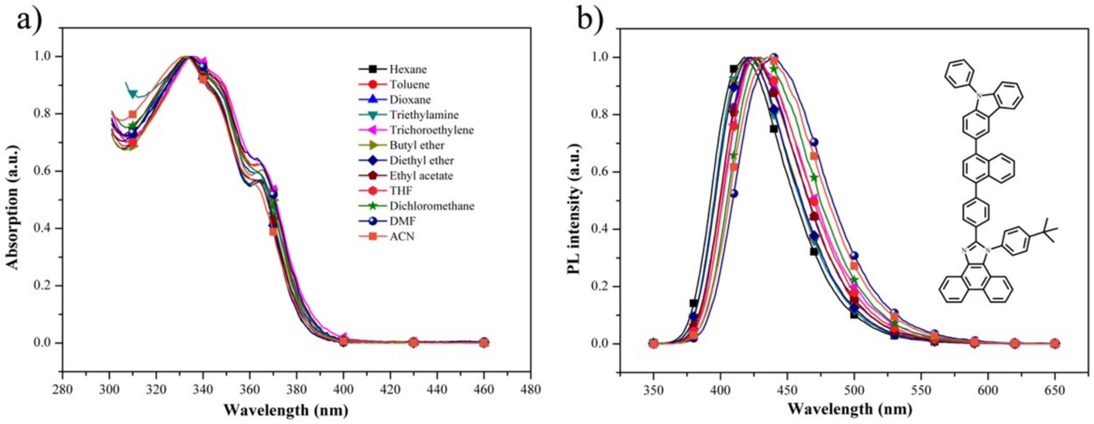

Fig. S4 Solvent-dependent absorption and PL spectra of TPINCz.

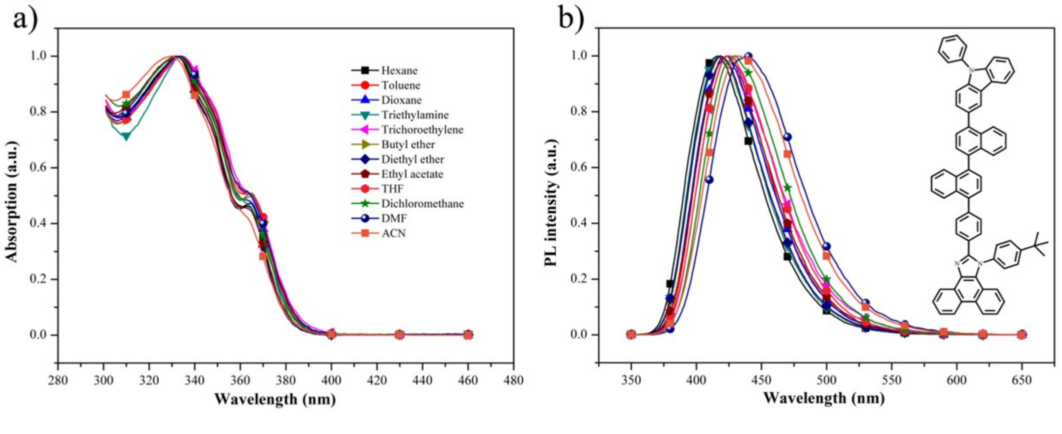

Fig. S5 Solvent-dependent absorption and PL spectra of TPIBNCz.

Table S1 Detailed photophysical data of TPIBCz, TPINCz and TPIBNCz in different solvents.

Solvent	ε[a]	n[b]	Δf[c]	TPIBCz			TPINCz			TPIBNCz		
				λ_{abs}[d] (nm)	λ_{pl}[e] (nm)	ν_a-ν_f[f] (cm^{-1})	λ_{abs}[d] (nm)	λ_{pl}[e] (nm)	ν_a-ν_f[f] (cm^{-1})	λ_{abs}[d] (nm)	λ_{pl}[e] (nm)	ν_a-ν_f[f] (cm^{-1})
n-hexane	1.89	1.3727	0.0008	336	412	5490.1	333	418	6106.6	332	416	6082.0
toluene	2.38	1.4969	0.0132	340	418	5488.3	336	422	6065.2	335	423	6210.1
1,4-dioxane	2.25	1.4203	0.0252	339	416	5460.1	335	422	6154.1	334	423	6299.5
triethylamine	2.42	1.401	0.0477	337	413	5460.5	333	421	6277.1	333	419	6163.7
trichoroethylene	3.39	1.474	0.0878	340	420	5602.2	336	425	6232.5	334	423	6299.5
butyl ether	3.08	1.399	0.0957	337	414	5519.0	334	422	6243.4	333	419	6163.7
diethyl ether	4.33	1.352	0.1669	336	413	5548.8	332	423	6479.8	332	418	6197.0
ethyl acetate	6.02	1.372	0.1998	336	415	5665.5	333	426	6555.9	331	424	6626.6
THF	7.58	1.407	0.2096	338	417	5605.0	335	428	6486.3	334	425	6410.7
dichoromethane	8.93	1.424	0.2172	338	420	5776.3	333	430	6774.2	332	429	6810.5
DMF	36.70	1.431	0.2742	337	421	5920.6	335	440	7123.5	334	439	7161.1
ACN	36.64	1.344	0.3050	334	418	6016.6	332	435	7132.0	330	432	7154.9

[a] Dielectric constant. [b] Refractive index. [c] Orientation polarization of solvents, calculated from $\Delta f = (\varepsilon-1)/(2\varepsilon+1)-(n^2-1)(2n^2+1)$. [d] Absorption and [e] fluorescent emission maximum. [f] Stokes shift.

Single carrier-only devices

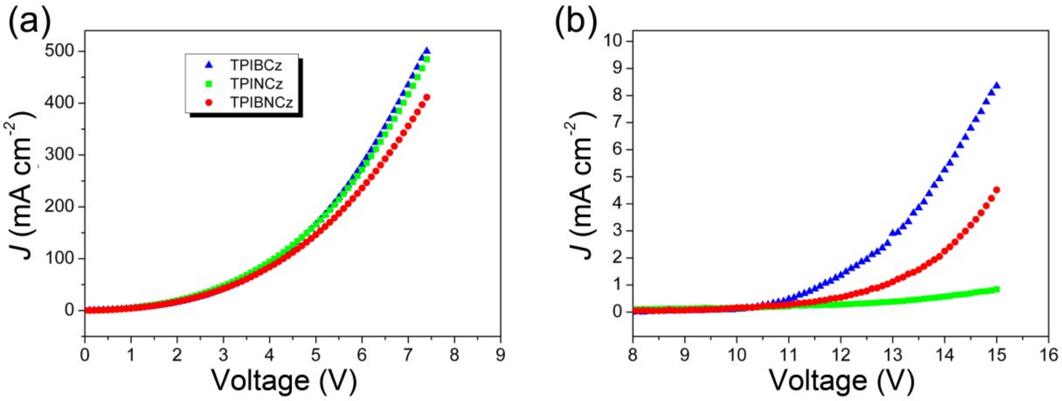

Fig. S6 Current density-voltage characteristics of the TPIBCz, the TPINCz and the TPIBNCz based (a) hole-only devices and (b) electron-only devices.

EL versus PL spectra

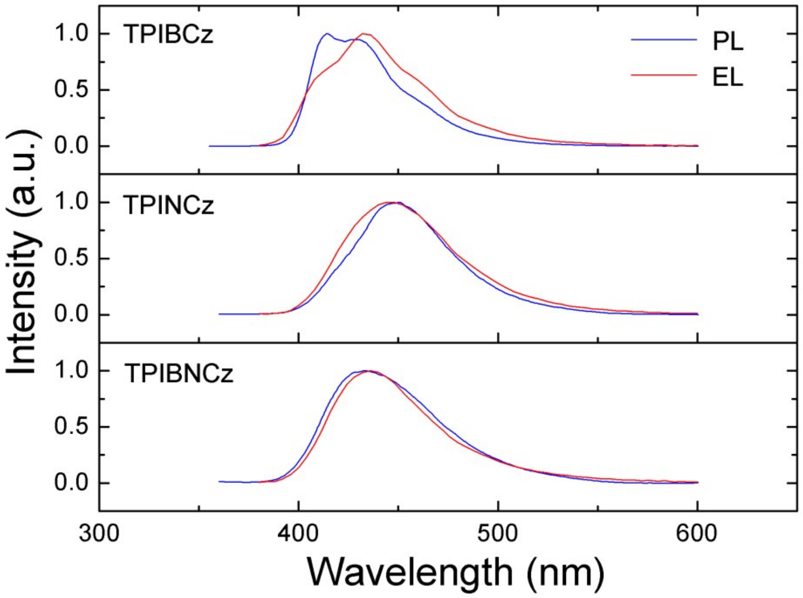

Fig. S7 Electroluminescence spectra of non-doped OLEDs detected at 1000 cd m^{-2} and photoluminescence spectra of the thin films prepared on clean quartz by thermal evaporation.

TD-DFT calculation

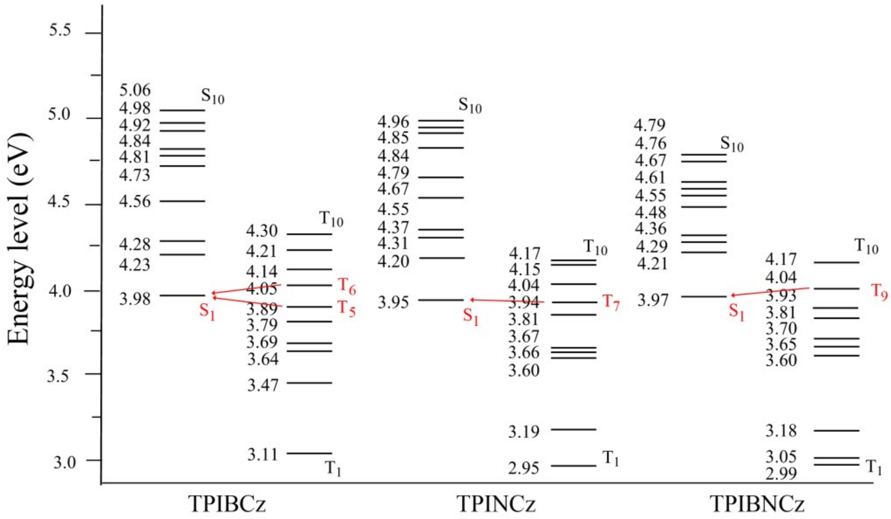

Fig. S8 Energy levels of first-ten singlet/triplet excited states from TD-DFT calculation. Red arrows indicate the potential RISC channels.

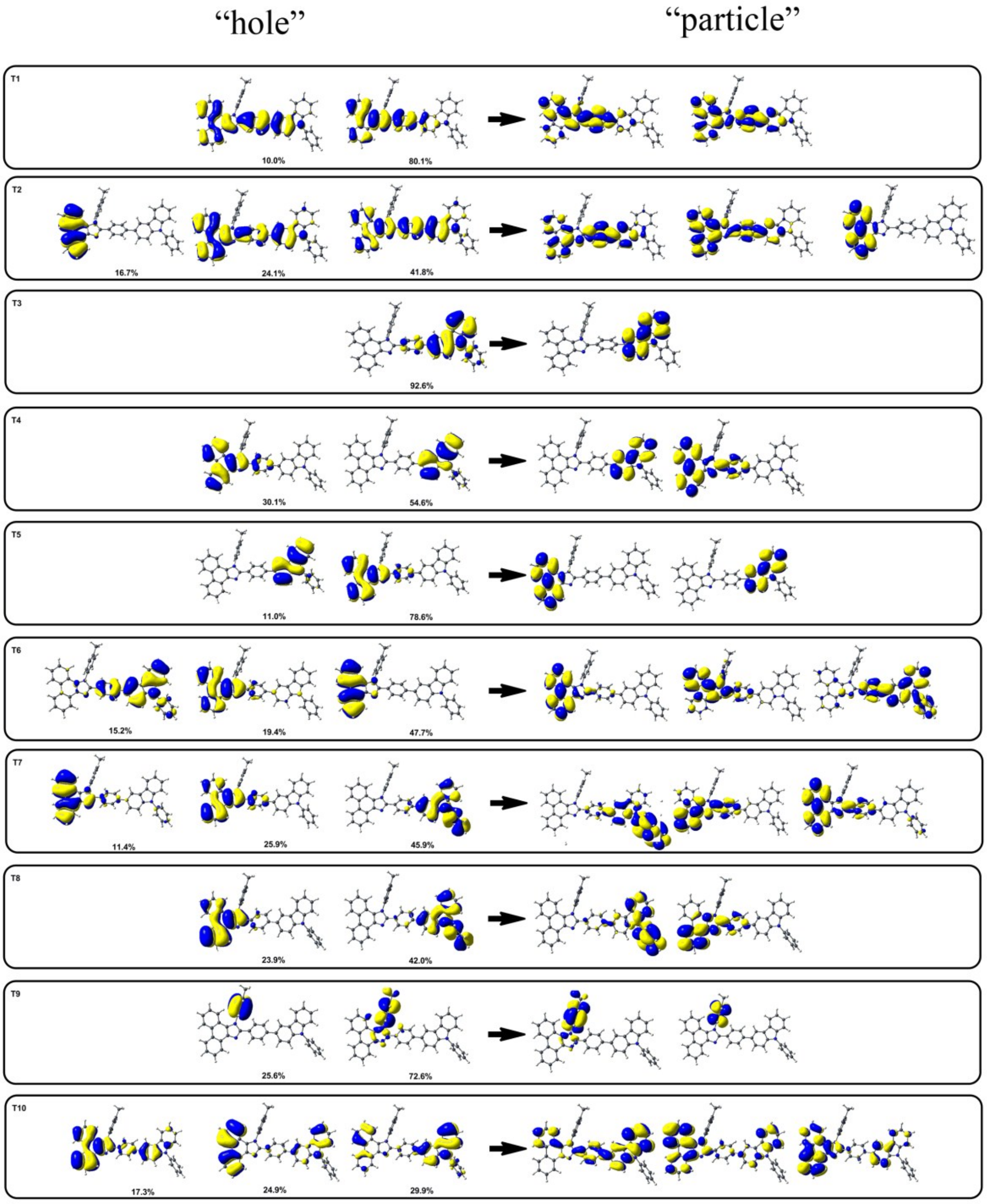

Fig. S9 First-ten NTOs of triplet excited states of TPIBCz.

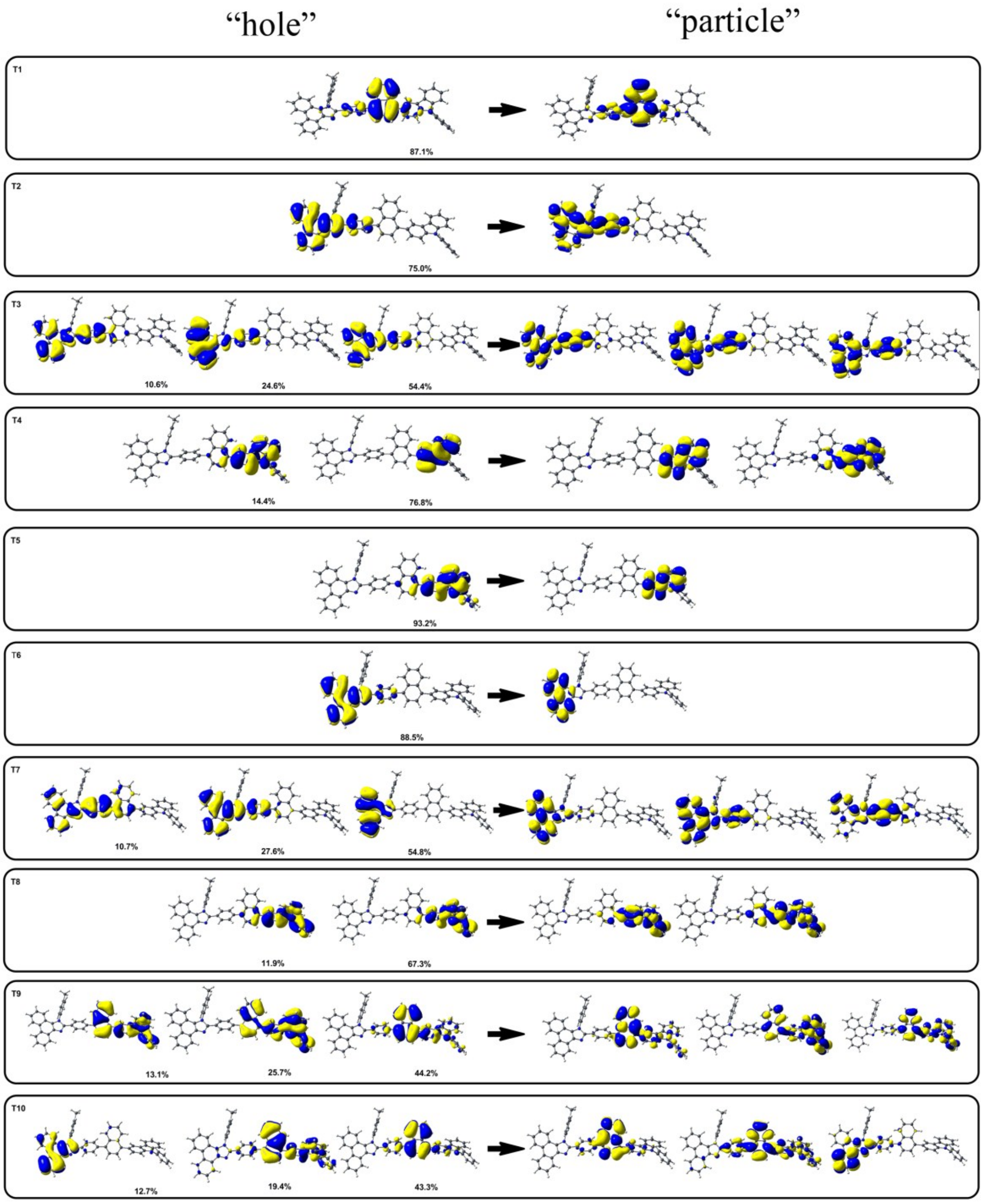

Fig. S10 First-ten NTOs of triplet excited states of TPINCz.

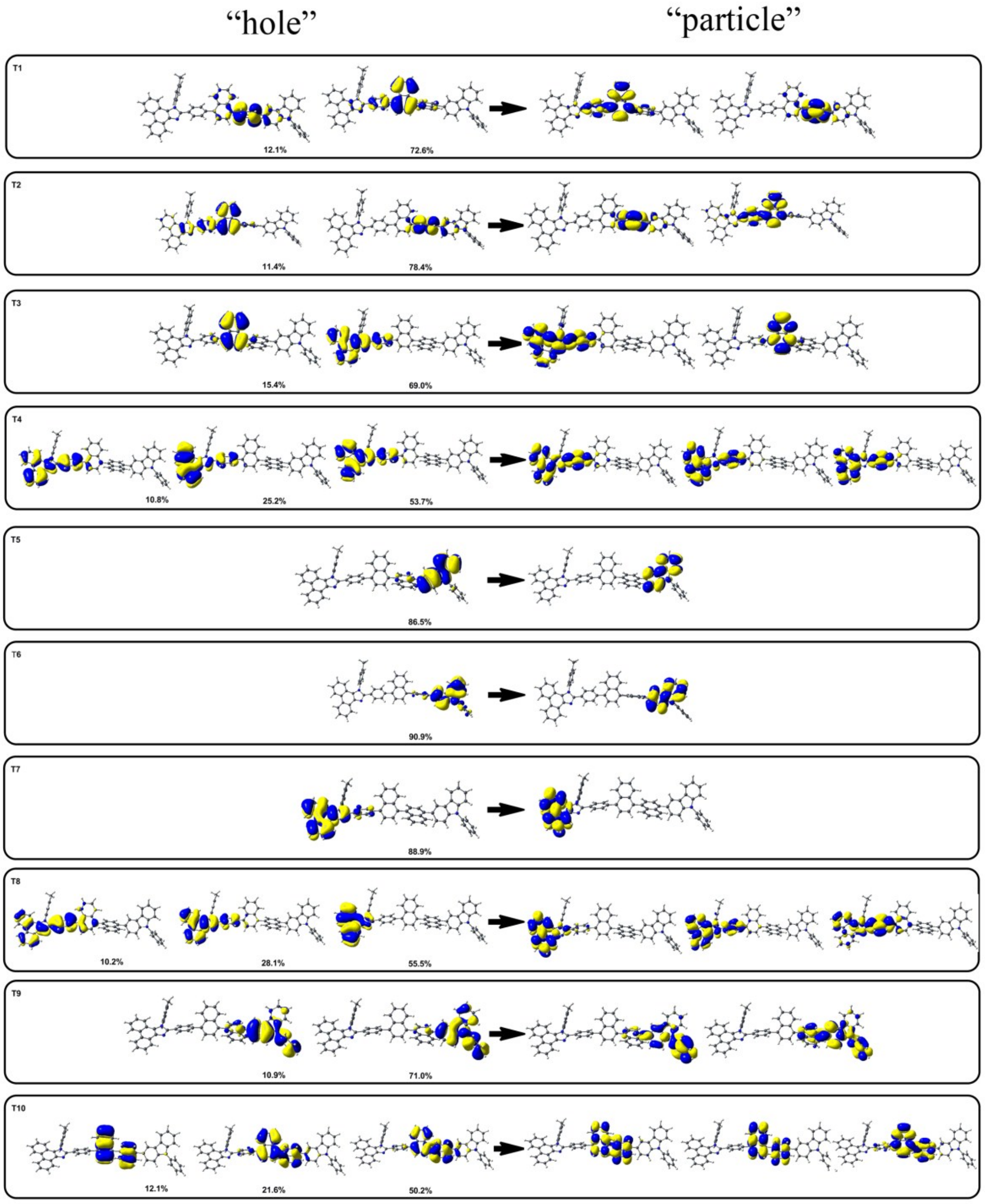

Fig. S11 First-ten NTOs of triplet excited states of TPIBNCz.

Luminance-current density characteristics

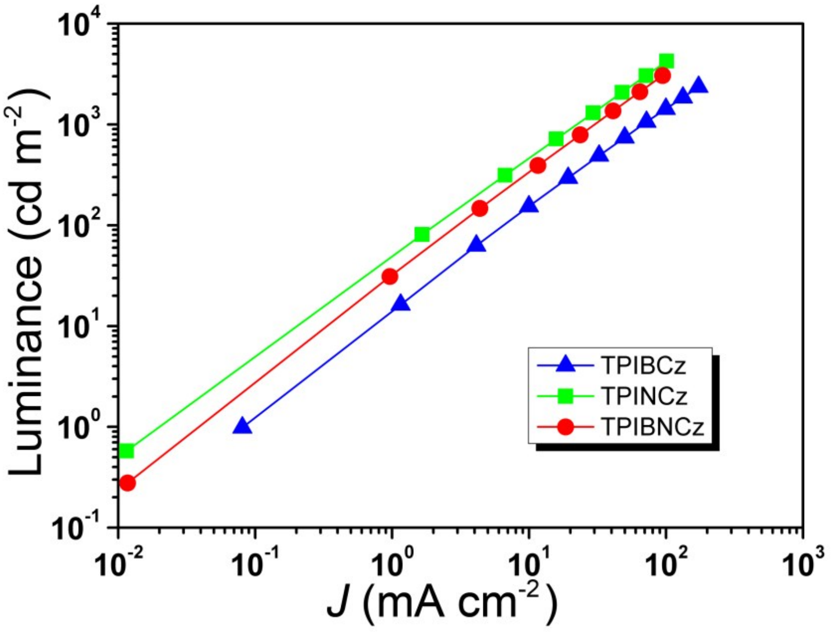

Fig. S12 Luminance-current density characteristics of the TPIBCz, the TPINCz and the TPIBNCz-based non-doped OLEDs.

CIE map

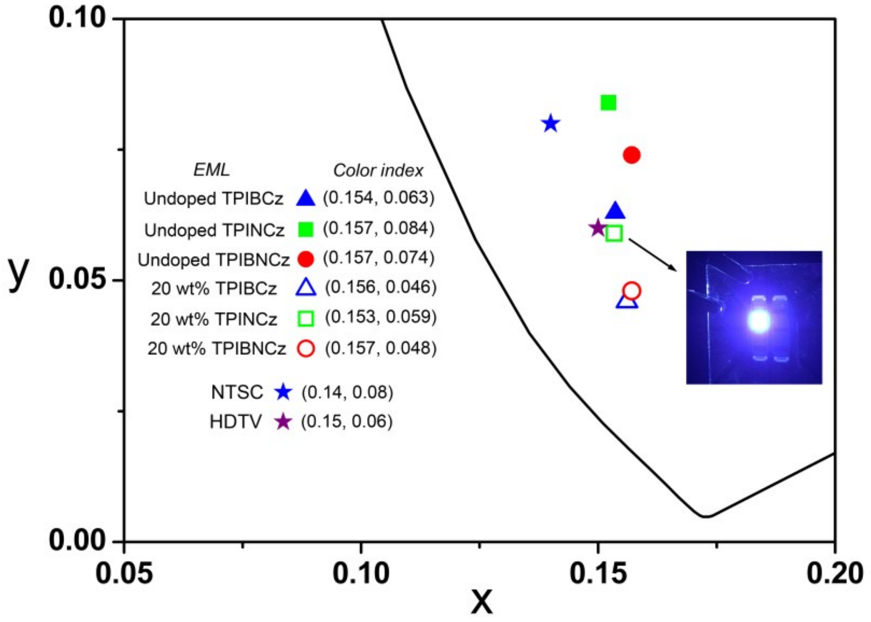

Fig. S13 CIE coordinates of the fabricated OLEDs.

Current density-voltage-luminance characteristics

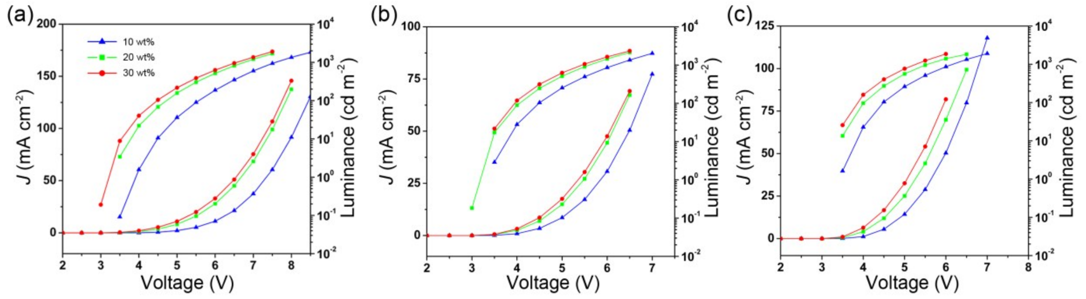

Fig. S14 Current density-voltage-luminance curves of the CBP-doped devices based on (a) TPIBCz, (b) TPINCz and (c) TPIBNCz, respectively.

Performance comparison

Table S2. Key performance data for the devices in this work and other high efficiency deep-blue OLEDs with $CIE_y \leq 0.08$.

Emitter	V_{on} (V)	λ_{EL} (nm)	$CIE_{x,y}$	EQE[d] (%)	EQE roll-off[e] (%)	Ref.[g]
SiPIM[a]	4.2	420	(0.163, 0.040)	6.29/ 4.72/ ~3[c]	> 50 [c]	4 (27)
TDAF[a]	2.5	-	(0.158, 0.041)	5.3/ -/ -	-	5
TPA-TAZ[a]	~3.1[c]	-	(0.158, 0.043)	6.8/ -/ 5.38	20.9	6 (31)
TPIBCz[b]	3.3	432	(0.156, 0.046)	5.46/ 5.39/ 4.96	9.2	This work
TPIBNCz[b]	3.2	428	(0.157, 0.048)	5.99/ 5.95/ 5.47	8.7	This work
C3FLA-2[b]	3.4	-	(0.156, 0.048)	8.0/ 6.5/ 3.7	53.8	7 (59)
TPAXAN[a]	3.4	428	(0.155, 0.049)	4.62/ -/ -[f]	-[f]	8
m-TPA-PPI[a]	3.2	404	(0.161, 0.049)	3.33/ -/ 3.00[c]	9.9	9 (60)
TCPC-6[a]	-	425	(0.16, 0.05)	3.72/ -/ -	-	10
PIBCz[a]	2.7	414	(0.15, 0.05)	2.74/ 2.67/ ~2.10[c]	23.4	11 (61)
DPA-PIM[a]	3.2	436	(0.15, 0.05)	5.1/ 4.9/ 3.6	29.5	12 (62)
XBTPI[a]	3.1	428	(0.16, 0.05)	4.93/ 4.80/ 4.05	17.8	13 (63)
TTP-TPI[a]	3.1	424	(0.16, 0.05)	5.02/ -/ 3.98	20.7	14 (64)
CzS1[a]	3.5	426	(0.157, 0.055)	4.21/ 4.20/ 3.19	24.2	15 (23)
TPINCz[b]	3.1	440	(0.153, 0.059)	6.96/ 6.94/ 6.56	5.7	This work
BD3[b]	3.7	432	(0.15, 0.06)	12/ 5.3/ 4.2	65	16 (28)
M2[a]	-	428	(0.166, 0.056)	3.02/ -/ -	-	17
m-BBTPI[a]	3.2	428	(0.16, 0.06)	3.63/ 3.61/ 3.36	7.4	18 (24)
3[b]	2.8	-	(0.15, 0.06)	6.5/ 6.0/ 4.7	27.6	19 (33)
PyINA[b]	3.4	432	(0.156, 0.06)	5.05/ 5.05/ 4.67	7.5	20 (65)
TPIBCz[a]	3.0	435	(0.154, 0.063)	3.38/ 3.32/ 3.22	4.7	This work
3(DTC-DPS)[b]	-	423	(0.15, 0.07)	9.9/ -/ - [f]	- [f]	21
DPT-TPI[a]	2.9	432	(0.16, 0.07)	5.25/ -/ 4.62	12	14 (64)
POAn[a]	3.0	445	(0.15, 0.07)	4.7/ 4.5/ -	-	22
PPI-PPIPCz[a]	3.4	-	(0.15, 0.07)	8.1/ 6.8/ 6.0	25.9	23 (66)
TPA-PA[a]	3.8	428	(0.16, 0.073)	7.23/ -/ -	-	24
TPIBNCz[a]	3.2	436	(0.157, 0.074)	5.09/ 4.60/ 5.08	0.2	This work
PMSO[b]	3.2	445	(0.152, 0.077)	6.80/ 6.63/ 5.64	17.7	25 (32)
DPSF[b]	3.5	435	(0.15, 0.08)	5.41/ -/ < 3[c]	> 44[c]	26 (67)
BPCC[b]	4.0	416	(0.16, 0.08)	4.9/ -/ ~3.7	~ 27	27 (68)
BiPI-1[a]	2.8	440	(0.15, 0.08)	6.18/ -/ 5.78	6.5	28 (69)
TPINCz[a]	3.1	448	(0.157, 0.084)	5.95/ 5.95/ 5.83	2.0	This work

[a] Non-doped device. [b] Doped device. [c] Estimated from reference. [d] Efficiency at maximum, 100 and 1000 cd m^{-2}, respectively. [e] EQE roll-off at 1000 cd m^{-2}. [f] Not applicable due to maximum luminance < 1000 cd m^{-2} estimated from references. [g] reference numbers given in brackets are from the main text (corresponding to Figure 7).

References

1 D. F. Eaton, *Pure Appl. Chem.*, 1988, **60**, 1107–1114.

2 E. Faggi, R. M. Sebastián, R. Pleixats, A. Vallribera, A. Shafir, A. Rodríguez-Gimeno and C. Ramírez de Arellano, *J. Am. Chem. Soc.*, 2010, **132**, 17980–17982.

3 Y. Zhang, S.-L. Lai, Q.-X. Tong, M.-F. Lo, T.-W. Ng, M.-Y. Chan, Z.-C. Wen, J. He, K.-S. Jeff, X.-L. Tang, W.-M. Liu, C.-C. Ko, P.-F. Wang and C.-S. Lee, *Chem. Mater.*, 2012, **24**, 61–70.

4 Z. Gao, G. Cheng, F. Shen, S. Zhang, Y. Zhang, P. Lu and Y. Ma, *Laser Photonics Rev.*, 2014, **8**, L6–L10.

5 C.-C. Wu, Y.-T. Lin, K.-T. Wong, R.-T. Chen and Y.-Y. Chien, *Adv. Mater.*, 2004, **16**, 61–65.

6 A. Obolda, Q. Peng, C. He, T. Zhang, J. Ren, H. Ma, Z. Shuai and F. Li, *Adv. Mater.*, 2016, **28**, 4740–4746.

7 J.-H. Jou, S. Kumar, P.-H. Fang, A. Venkateswararao, K. R. J. Thomas, J.-J. Shyue, Y.-C. Wang, T.-H. Li and H.-H. Yu, *J. Mater. Chem. C*, 2015, **3**, 2182–2194.

8 R. Kim, S. Lee, K.-H. Kim, Y.-J. Lee, S.-K. Kwon, J.-J. Kim and Y.-H. Kim, *Chem. Commun.*, 2013, **49**, 4664–4666.

9 H. Liu, Q. Bai, L. Yao, H. Zhang, H. Xu, S. Zhang, W. Li, Y. Gao, J. Li, P. Lu, H. Wang, B. Yang and Y. Ma, *Chem. Sci.*, 2015, **6**, 3797–3804.

10 S. Tang, M. R. Liu, P. Lu, H. Xia, M. Li, Z. Q. Xie, F. Z. Shen, C. Gu, H. P. Wang, B. Yang and Y. G. Ma, *Adv. Funct. Mater.*, 2007, **17**, 2869–2877.

11 D. He, Y. Yuan, B. Liu, D.-Y. Huang, C.-Y. Luo, F. Lu, Q.-X. Tong and C.-S. Lee, *Dyes Pigments*, 2017, **136**, 347–353.

12 C. He, H. Guo, Q. Peng, S. Dong and F. Li, *J. Mater. Chem. C*, 2015, **3**, 9942–9947.

13 W.-C. Chen, Y. Yuan, G.-F. Wu, H.-X. Wei, L. Tang, Q.-X. Tong, F.-L. Wong and C.-S. Lee, *Adv. Opt. Mater.*, 2014, **2**, 626–631.

14 Y. Yuan, J.-X. Chen, F. Lu, Q.-X. Tong, Q.-D. Yang, H.-W. Mo, T.-W. Ng, F.-L. Wong, Z.-Q. Guo, J. Ye, Z. Chen, X.-H. Zhang and C.-S. Lee, *Chem. Mater.*, 2013, **25**, 4957–4965.

15 J. Ye, Z. Chen, M.-K. Fung, C. Zheng, X. Ou, X. Zhang, Y. Yuan and C.-S. Lee, *Chem. Mater.*, 2013, **25**, 2630–2637.

16 J.-Y. Hu, Y.-J. Pu, F. Satoh, S. Kawata, H. Katagiri, H. Sasabe and J. Kido, *Adv. Funct. Mater.*, 2014, **24**, 2064–2071.

17 Z. Gao, Y. Liu, Z. Wang, F. Shen, H. Liu, G. Sun, L. Yao, Y. Lv, P. Lu and Y. Ma, *Chem. – Eur. J.*, 2013, **19**, 2602–2605.

18 W.-C. Chen, G.-F. Wu, Y. Yuan, H.-X. Wei, F.-L. Wong, Q.-X. Tong and C.-S. Lee, *RSC Adv.*, 2015, **5**, 18067–18074.

19 I. Kondrasenko, Z.-H. Tsai, K. Chung, Y.-T. Chen, Y. Y. Ershova, A. Doménech-Carbó, W.-Y. Hung, P.-T. Chou, A. J. Karttunen and I. O. Koshevoy, *ACS Appl. Mater. Interfaces*, 2016, **8**, 10968–10976.

20 T. Shan, Y. Liu, X. Tang, Q. Bai, Y. Gao, Z. Gao, J. Li, J. Deng, B. Yang, P. Lu and Y. Ma, *ACS Appl. Mater. Interfaces*, 2016, **8**, 28771–28779.

21 Q. Zhang, J. Li, K. Shizu, S. Huang, S. Hirata, H. Miyazaki and C. Adachi, *J. Am. Chem. Soc.*, 2012, **134**, 14706–14709.

22 C.-H. Chien, C.-K. Chen, F.-M. Hsu, C.-F. Shu, P.-T. Chou and C.-H. Lai, *Adv. Funct. Mater.*, 2009, **19**, 560–566.
23 C. Li, S. Wang, W. Chen, J. Wei, G. Yang, K. Ye, Y. Liu and Y. Wang, *Chem. Commun.*, 2015, **51**, 10632–10635.
24 S. Tang, W. Li, F. Shen, D. Liu, B. Yang and Y. Ma, *J. Mater. Chem.*, 2012, **22**, 4401–4408.
25 X. Tang, Q. Bai, Q. Peng, Y. Gao, J. Li, Y. Liu, L. Yao, P. Lu, B. Yang and Y. Ma, *Chem. Mater.*, 2015, **27**, 7050–7057.
26 X. Xing, L. Xiao, L. Zheng, S. Hu, Z. Chen, B. Qu and Q. Gong, *J. Mater. Chem.*, 2012, **22**, 15136–15140.
27 Y.-H. Chung, L. Sheng, X. Xing, L. Zheng, M. Bian, Z. Chen, L. Xiao and Q. Gong, *J. Mater. Chem. C*, 2015, **3**, 1794–1798.
28 Z.-L. Zhu, M. Chen, W.-C. Chen, S.-F. Ni, Y.-Y. Peng, C. Zhang, Q.-X. Tong, F. Lu and C.-S. Lee, *Org. Electron.*, 2016, **38**, 323–329.
